# Activated molecular probes for enzyme recognition and detection

**DOI:** 10.7150/thno.66676

**Published:** 2022-01-01

**Authors:** Meng Yuan, Ying Wu, Caiyan Zhao, Zhongxiang Chen, Lichao Su, Huanghao Yang, Jibin Song

**Affiliations:** MOE key laboratory for analytical science of food safety and biology Institution, College of Chemistry, Fuzhou University, Fuzhou 350108, China

**Keywords:** enzyme, biosensor, molecular probe, photoacoustic imaging, fluorescence imaging (FLI)

## Abstract

Exploring and understanding the interaction of changes in the activities of various enzymes, such as proteases, phosphatases, and oxidoreductases with tumor invasion, proliferation, and metastasis is of great significance for early cancer diagnosis. To detect the activity of tumor-related enzymes, various molecular probes have been developed with different imaging methods, including optical imaging, photoacoustic imaging (PAI), magnetic resonance imaging, positron emission tomography, and so on. In this review, we first describe the biological functions of various enzymes and the selectively recognized chemical linkers or groups. Subsequently, we systematically summarize the design mechanism of imaging probes and different imaging methods. Finally, we explore the challenges and development prospects in the field of enzyme activity detection. This comprehensive review will provide more insight into the design and development of enzyme activated molecular probes.

## Introduction

Enzymes are important biological catalysts that participate in various biological and physiological processes. Enzymes can promote chemical reactions in biological tissues under extremely mild conditions, and are essential for maintaining normal body functions. Therefore, the expression level of enzymes in organisms is closely related with the overall function of the biological system 1. In previous studies, it has been shown that abnormal enzyme activity in organisms is closely related with a variety of diseases. For example, matrix metalloproteinases (MMPs) are involved in the invasion, proliferation, and metastasis of malignant tumors.^2^ Furthermore, the highly active enzyme caspase-3 has been shown to be an important biomarker for apoptosis and cervical cancer 3. Therefore, real-time monitoring of enzyme activity and enzyme biodistribution in organisms is essential for identifying enzyme function, and for providing favorable conditions for early diagnosis and treatment. However, the complex and dynamic microenvironment poses significant challenges for the detection of enzyme activity *in vivo*.

Molecular imaging, as a non-invasive imaging method, is widely used in the visual detection of specific functional molecules in biological processes 4. Molecular imaging can help to detect abnormal enzyme expression before changes in the morphology of diseased tissue occur, reveal enzyme-related functions, and aid in early diagnosis of diseases, and has been considered a promising method for detecting enzyme activity *in vivo*. Molecular imaging requires corresponding imaging probes that can be activated by specific enzymes to generate analytical signals 5. Therefore, the development of highly specific and high-resolution imaging probes is essential for accurate detection of enzyme activity. To this end, a series of imaging probes have been constructed to detect enzyme activity *via* various mechanisms, including fluorescence resonance energy transfer (FRET), self-assembly, self-elimination, macrocyclization, and intramolecular charge transfer (ICT). For example, Yang *et al*., constructed a ratiometric fluorescent (FL) probe for the detection of caspase-3 activity via the FRET mechanism between Au and modified SiO_2_ nanoparticles 6. In addition, Xing *et al*. used the principle of cathepsin B (CTB) to trigger the *in situ* self-assembly of rare earth nanoparticles to achieve imaging detection at the tumor site 7. The construction of enzyme-activated probes opens a new avenue for early diagnosis and effective treatment of malignant tumors 8. However, to date, no comprehensive review is available that systematically and timely describes the progress in the attractive field of enzyme activity detection through responsive probes.

**Scheme 1:**
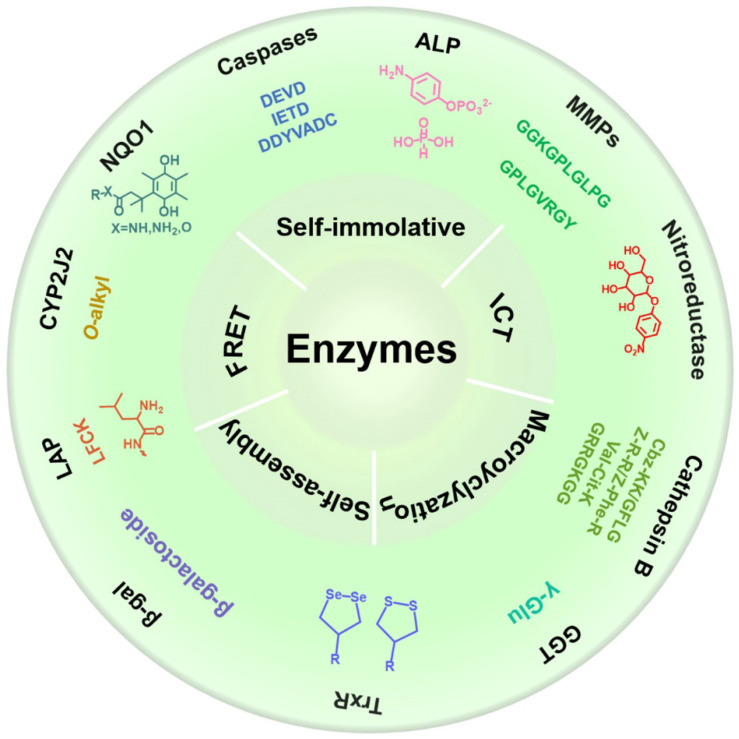
Schematic illustration of various activatable molecular probes for the detection of enzyme activity, and their applications in biomedical imaging.

In this review, we systematically summarize recent progress in the development of various activated molecular probes for the detection of enzyme activity, and describe their applications in biomedical imaging (**Scheme 1**). First, we describe the biological functions of various cancer-related enzymes and the chemical groups that are involved in selective recognition to better use the recognition moiety for designing a reasonable imaging probe for accurately detecting enzyme activity. Subsequently, we introduce the design strategy of the imaging probe and the underlying mechanism involved in signal activation. Furthermore, we discuss several mechanisms applied in the designing process of responsive probes, including FRET, ICT, self-elimination, macrocyclization, and self-assembly. Finally, we discuss in detail several typical imaging methods, including FL imaging (FLI), photoacoustic imaging (PAI), chemiluminescence (CHL) imaging, magnetic resonance imaging (MRI), positron emission tomography (PET) imaging, and photoluminescence imaging (PLI) that are associated with enzyme-activated molecular probes and the application of these imaging probes in the process of cancer diagnosis (**Table 1**). Overall, in this review, we systematically summarize enzyme-specific recognition groups in cancer diagnosis and provide guidance for future studies on the detection of cancer-related enzyme activity.

## 2. Peptide/group selective to enzymes

### 2.1. Caspase-selective peptides

Caspase-3/7 belongs to the class of cysteine proteases that exist in the cytoplasm and are important markers of cell apoptosis. Caspase-3/7 can be activated by the initiator caspase-8/9 only in the process of apoptosis 9. In normal cells, caspase-3/7 is not activated. Activated caspase-3/7 can hydrolyze part of proteins and further promote tumor apoptosis. Thus, high expression levels of caspase-3/7 in cancer cells can be used as a specific biomarker for apoptosis, and accurate detection of caspase-3/7 activity can provide important information for early diagnosis of tumors and the appropriate selection of anticancer drugs.

#### 2.1.1. The DEVD sequence

**Figure 1:**
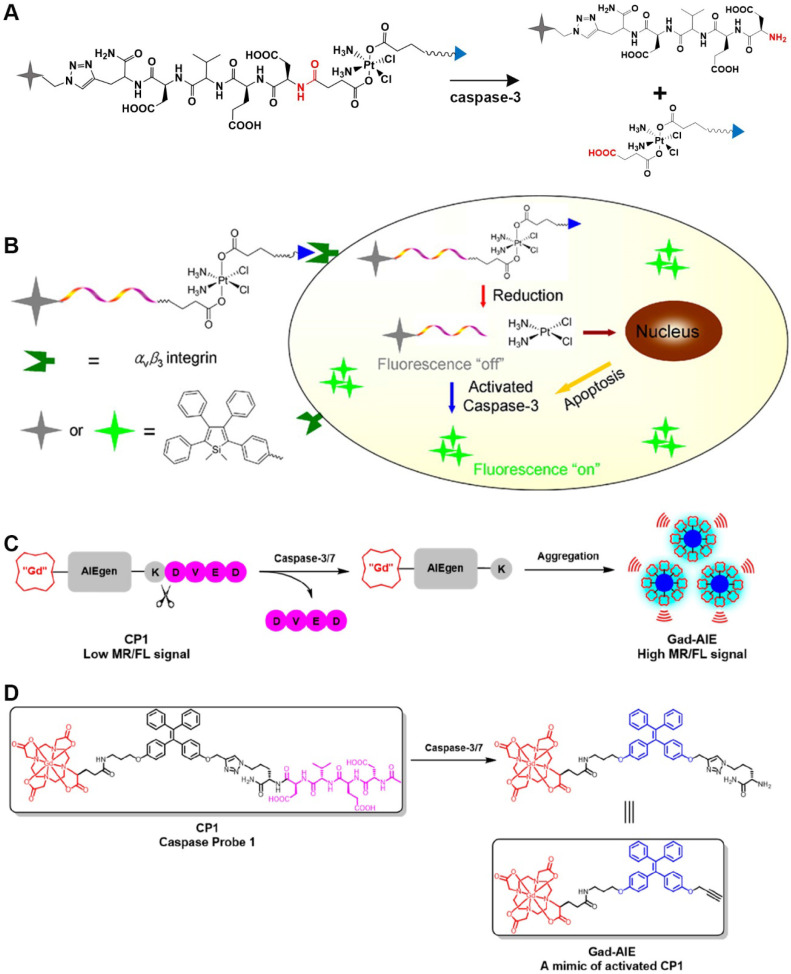
(A) Schematic illustration of the stimulation-response mechanism of caspase-3 toward the DEVD peptide; (B) Illustration of the FL probe responsiveness to caspase-3; (C) Schematic illustration of stimulation-response mechanism of caspase-3/7 to the DEVD peptide; (D) Illustration of the PA probe activated by caspase-3/7; (B) Reproduced with permission 10. Copyright 2014, American Chemical Society. (C and D) Reproduced with permission 11. Copyright 2019, American Chemical Society.

The peptide sequence Asp-Glu-Val-Asp (DEVD), as a recognition peptide fragment of two caspases (caspase-3 and caspase-7), can be cleaved by caspase-3/7 to release free amino groups. Both caspase-3 and caspase-7 have the same recognition sites in the DEVD peptide. Bases on this feature, a series of biological probes have been designed for the detection of caspase-3/7 activity. For example, Liu *et al*., used an aggregation-induced emission (AIE) molecule to prepare a light-activated apoptosis sensor 10. In this probe, a caspase-3-sensitive DEVD sequence was used to achieve a responsive release of FL dyes (**Figure 1A**). Caspase is a protease with a cysteine residue in the active site that hydrolyzes the amide bond at the end of aspartic acid. Figure 1A shows that caspase-3 cleaves the DEVD peptide to release the AIE molecule and the cisplatin prodrug. In addition, anti-cancer drugs in the probe can be effectively delivered to the tumor site via c-RGD-targeting peptides for selective treatment of diseased tissue (**Figure 1B**). In another study, a caspase-3/7-activated dual-mode imaging probe was designed using AIE molecules to observe the cellular apoptosis process 11. Using this method, a hydrophilic peptide was covalently linked to an AIE molecule that was chelated with Gd^3+^ (**Figure 1C**). Based on the hydrophobic-hydrophobic interaction between AIE molecules, and after the enzymatic response, free AIE molecules aggregated and self-assembled to emit a FL signal. At the same time, a large amount of Gd^3+^ aggregated because of the occurrence of self-assembly, which accordingly enhanced the magnetic resonance signal (**Figure 1D**).

### 2.2. MMP-selective peptides

MMPs are endopeptidases that rely on zinc or calcium to express their catalytic activity. They are involved in a series of physiological processes, including degradation, extracellular matrix remodeling, tissue reorganization, and the release of bioactive molecules. In the MMP family, matrix metalloproteinase-2 and matrix metalloproteinase-9 (MMP-2/9) have been considered important markers for diseases, such as inflammation, cardiovascular diseases, and cancer 12. In general, in normal cells, MMP activities are low, however, in tumor tissue, MMP-2/9 are significantly overexpressed. MMP-active peptides have been used to accurately detect the biological distribution, and the activity of cancer-related MMPs has been described 13.

#### 2.2.1. GPLGVRGY sequence

**Figure 2:**
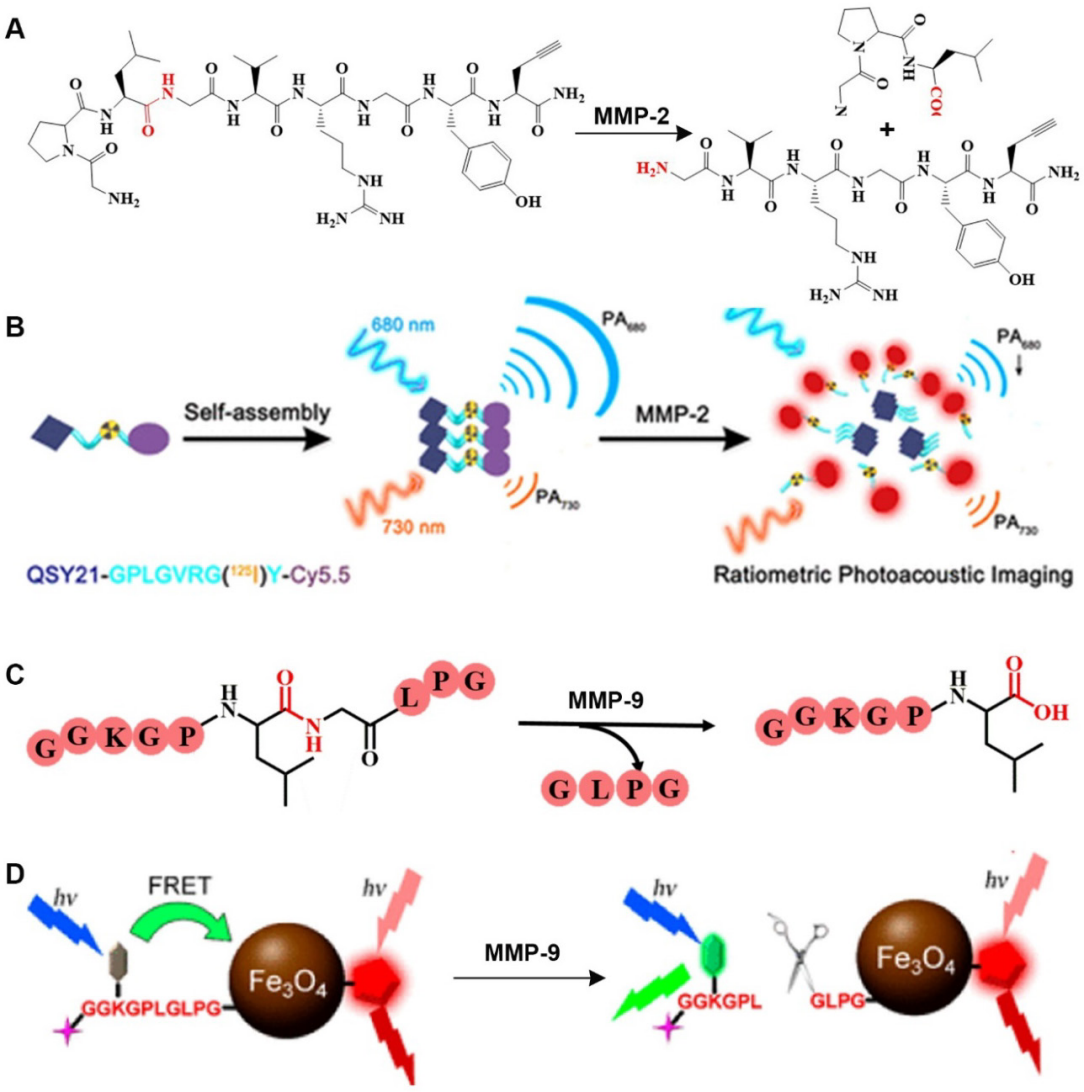
(A) Schematic illustration of stimulation-response mechanism of MMP-2 to GPLGVRGY peptide; (B) Illustration of the FL probe responsive by MMP-2; (C) Schematic illustration of the stimulation-response mechanism of MMP-9 to GGKGPLGLPG peptide; (D) Illustration of the FL probe responsive by MMP-9; (B) Reproduced with permission 14. Copyright 2019, American Chemical Society. (D) Reproduced with permission 15. Copyright 2017, American Chemical Society.

After being activated by an enzyme, GPLGVRGY, a sensitive peptide of MMP-2, can be cleaved into two fragments. Because the GPLGVRGY peptide has a good biocompatibility, it can be combined with some hydrophobic small molecules to bring these small molecules into the aqueous phase. In addition, different particles (small organic molecules or inorganic nanoparticles) can be combined through the GPLGVRGY peptide to prepare responsive bioprobes. For example, based on the ability of MMP-2 to selectively recognize and cleave the GPLGVRGY peptide sequence peptide sequence, a ratiometric PA probe has been constructed (**Figure 2A**) 14, consisting of a near-infrared (NIR) FL dye (Cy5.5) and a FL quencher (QSY21) that is covalently linked to a biocompatible peptide substrate. After MMP-2 stimulates cleavage, the aggregation state of the FL dye and quencher in the probe changes, causing the probe to exhibit a significant MMP-2 concentration-dependent absorption at around 680 nm, while that at around 730 nm was MMP-2 concentration independent (**Figure 2B**). Accordingly, the MMP-2 concentration is quantitatively detected in live breast cancer cells by recording changes in the PA signal at different wavelengths.

#### 2.2.2. GGKGPLGLPG sequence

MMP-9 recognizes a specific peptide sequence. For example, MMP-9 can selectively cleave the peptide GGKGPLGLPG into two peptides, GGKGPL and GLPG (**Figure 2C**). Based on this characteristic, Gao *et al*., designed a dual-ratiometric FL probe for detecting MMP-9 activity in tumors 15. In this probe, Fe_3_O_4_ nanoparticles are linked to a pH-sensitive dye via a biocompatible peptide to establish a FRET system to sensitize the pH of the tumor microenvironment. A FL dye (Cy5.5) was used as a reference to create a dual-ratiometric FL probe (**Figure 2D**). This dual-ratiometric imaging probe quantitatively revealed that the overexpression of MMP-9 at tumor sites and the spatial heterogeneity of abnormal microenvironmental pH and synergistically guide *in vivo* tumor invasion in a mouse model of colon cancer.

### 2.3. Alkaline phosphatase (ALP)-selective groups

ALP is a serum marker, widely distributed in mammals, which can catalyze the dephosphorylation of nucleic acid proteins and small molecules 16. Abnormal serum levels of ALP are often closely associated with certain pathological processes, such as diabetes, breast cancer, prostate cancer, liver cancer, and other diseases. ALP has been considered an important biomarker in clinical diagnosis. To increase therapeutic efficacy, different ALP detection strategies have been reported, such as electrochemical methods, chromatography, surface-enhanced resonance Raman scattering, colorimetry, FL detection, and electrochemical luminescence.

**Figure 3:**
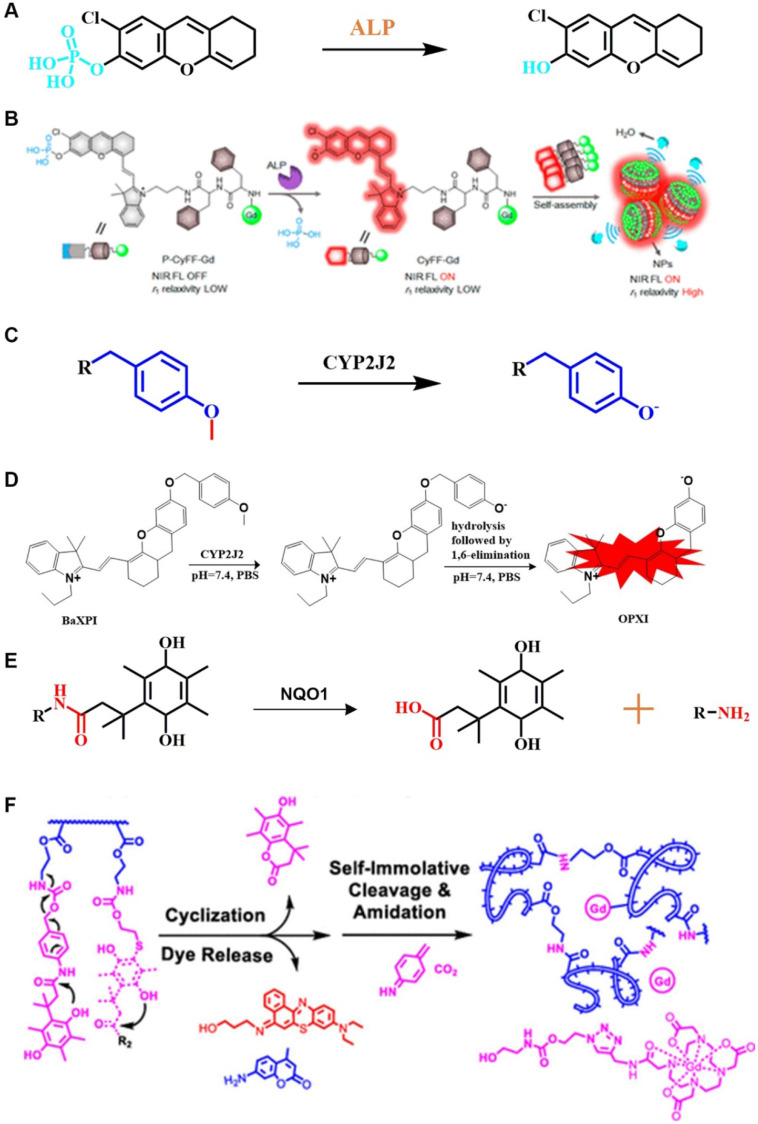
(A) Schematic illustration of the stimulation-response mechanism of ALP to phosphate groups; (B) Illustration of FL probes that are responsive to ALP; (C) Schematic illustration of the stimulation-response mechanism of CYP2J2 to the O-alkyl group; (D) Illustration of FL probes that are responsive to CYP2J2; (E) Mechanism of recognition of quinone propionic acid derivatives by NQO1; (F) Schematic diagram of NQO1-triggered release of FL molecules; (B) Reproduced with permission 40. Copyright 2019, American Chemical Society. (D) Reproduced with permission 86. Copyright 2018, American Chemical Society. (F) Reproduced with permission 92. Copyright 2020, American Chemical Society.

The phosphate group is a recognition group for ALP. ALP can catalyze the dephosphorylation of molecules containing free phosphate groups, thereby changing the properties of these molecules. Using the characteristics of ALP to remove phosphate groups from chemical structures (**Figure 3A**), FL-controllable imaging probes can be created. ALP can catalyze the dephosphorylation of molecules containing phosphate groups, change the water solubility and luminescence properties of dye molecules, and provide ideas for designing FL probes for detecting ALP activity. Ye *et al*. designed an enzyme-triggered membrane-localized self-assembling probe, P-CyFF-Gd, which targets ALP (**Figure 3B**) 40. P-CyFF-Gd combines FL and magnetic resonance imaging to monitor enzyme activity and spatial location *in vivo*.

### 2.4. Cytochrome P450 2J2 (CYP2J2)-selective groups

As an oxidative metabolism enzyme for various endogenous and exogenous compounds, CYP2J2 can metabolize polyunsaturated fatty acids and generate important signal molecules, leading to the formation of epoxyeicosatrienoic acids (EETs), and ultimately promote cellular proliferation 83. CYP2J2 is highly expressed in malignant tumors, and a significant increase in CYP2J2 activity greatly promotes the proliferation of cancer cells and reduces the rate of apoptosis 84. Moreover, because CYP2J2 produces several pro-angiogenic lipid products during metabolic responses, it accelerates cancer cell metastasis by promoting angiogenesis around and inside the tumor 85. Therefore, using CYP2J2 as a biomarker to detect the activity and localize CYP2J2 *in vivo* is of great significance for early diagnosis and treatment of cancer.

#### 2.4.1. O-alkyl group

O-alkyl, as a specific recognition moiety of CYP2J2, undergoes O-demethylation after being triggered by activated CYP2J2 (**Figure 3C**). According to the molecular reaction principle, O-alkyl, a self-immolative linker, and an NIR FL molecule are covalently coupled. By shortening the distance between the metabolic moiety and the catalytic center of the enzyme, Ma *et al*. successfully constructed a FL probe that is capable of real-time detection of CYP2J2 activity (**Figure 3D**) 86.

### 2.5. NAD(P)H: quinone oxidoreductase isozyme 1 (NQO1)-selective linkers

NQO1 is a cytosolic flavinase that can reduce quinone and its derivatives to a less toxic hydroquinone. In brief, nicotinamide adenine dinucleotide phosphate (NADPH) and nicotinamide adenine dinucleotide (NADH) were used as electron donors to for quinone compounds to undergo a mandatory two-electron reduction reaction to protect cells from oxidative stress 87. In previous studies, it was shown that the expression level of NQO1 was significantly upregulated (~5-200-fold) in several human malignancies, including breast cancer, cervical cancer, colon cancer, and liver cancer 88. Therefore, NQO1 has become an important marker for early diagnosis of a variety of diseases 89.

As a cytosolic flavinase, NQO1 can easily reduce the recognition group of quinone and its derivatives, thereby leading to intramolecular lactonization to form hydroquinone compounds 90. Notably, NQO1 has shown high selectivity and sensitivity to quinones (**Figure 3E**) 91. In general, NQO1-responsive quinone derivatives are covalently linked to FL dyes to establish a photo-induced electron transfer effect (PIETE), causing FL quenching of the dye. Upon an enzyme-mediated trigger, quinone derivatives are reduced to undergo intramolecular lactonization, and release FL dyes, resulting in a "turn off-turn on" response toward FL. By taking advantage of the selective reduction properties, NQO1-activated NIR FL probes were prepared by constructing a photosensitizer-conjugated polymer vesicle, which realizes the synergistic effect of NQO1 triggers on NIR FL emission and photodynamic therapy (PDT) (**Figure 3F**) 92. This integrated method of diagnosis and treatment is more practical than diagnosis and treatment of cancer in a single mode.

### 2.6. γ-glutamyltranspeptidase (GGT)-selective linkers/groups

GGT is a cell surface-related enzyme that can selectively catalyze the cleavage of glutathione to produce cysteyl-glycine, which is induced by plasma membrane dipeptidase to form cysteine and glycine. Because cysteine is closely related with malignant tumors, the metabolism of extracellular GSH triggered by GGT can provide favorable conditions for the growth and survival of cancer cells 93. In fact, it has previously been shown that GGT is overexpressed in some malignancies, such as ovarian, liver, and colon cancer 94. Therefore, GGT activity has been considered to be an important parameter for the diagnosis of related diseases 95.

**Figure 4:**
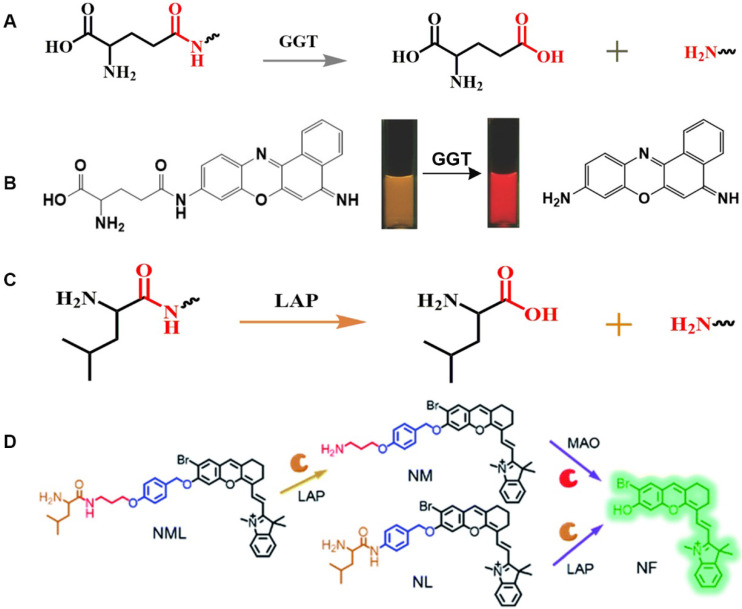
(A) Schematic illustration of the stimulation-response mechanism of GGT to γ-Glu; (B) Illustration of CV-Glu responsive to GGT; (C) Schematic illustration of the stimulation-response mechanism of LAPs to L-leucine; (D) Illustration of the FL probe responsive to LAPs; (B) Reproduced with permission 96. Copyright 2017, WILEY-VCH. (D) Reproduced with permission 103. Copyright 2019, The Royal Society of Chemistry.

γ-glutamine can specifically be catalyzed by GGT, and a series of responsive probes containing γ-glutamyl groups have been constructed (**Figure 4A**) 53. In general, a glutamine-containing compound is covalently coupled to FL molecules, and the GGT activity is observed in real time by changing the FL intensity at different wavelengths before and after the response. The enzyme-sensitive ratiometric FL probe cresyl violet-glutamic acid derivative (CV-Glu), a representative example, is constructed by directly covalently linking CV and Glu (**Figure 4B**) 96. CV-Glu is a ratiometric FL probe because CV-Glu and CV (products when CV-Glu is cleaved by GGT) have bright FL at two wavelengths (580 and 625 nm, respectively). By combining the ratiometric FL probe CV-Glu and the antibody-conjugated quantum dot probe, the cancerous, proliferative, and adenoma regions in fresh colon tissue can be accurately displayed 97.

### 2.7. Leucine aminopeptidase (LAP)-selective linkers/groups

LAPs are a class of metal peptidases that hydrolyze leucine residues at the amino terminus of a protein or peptide substrate 59. LAPs are involved in various physiological processes, including hydrolysis of biologically active peptides 98, degradation of DNA 99, and interaction with peptide-dependent signals 100. LAPs play a vital role in normal functioning of the body 56. Abnormal expression or altered activity of LAPs can lead to tumor invasion 101. In fact, overexpression of LAPs has been observed in various human diseases, including hepatocellular carcinoma, breast cancer, and endometrial cancer 99, thereby indicating that LAPs are a potential tumor predictive marker 102.

The N-terminus of the biocompatible peptide chain and the leucine amide bond formed between L-leucine and other amino compounds can be used as the recognition group for LAPs (**Figure 4C**). The highly selective and sensitive FL probes designed for the detection of LAPs usually adopt the following strategy: the L-leucine amide bond is formed between L-leucine and a chromophore by using a fluorophore as a chromophore. LAP enzyme-responsive recognition cleaves the L-leucine amide bond and enables the regulation of fluorophore luminescence. Based on the above-mentioned design principle, an enhanced “double-lock” and enzyme-activated FL probe was successfully constructed (**Figure 4D**) 103. The probe directly couples the response unit to the chromophore and shows weak or no FL, which is attributed to intermolecular or intramolecular FRET, PET, ICT, and other kinds of mechanism. After the enzyme triggers lysis, the FL signal is restored and the enzyme activity of the diseased tissue is accurately detected.

### 2.8. CTB-selective peptides

Cathepsins, members of the family of papain-like cysteine proteases, degrade lysosomal proteins in cells 104. Within the cathepsin family, CTB is considered essential because of its involvement in a variety of biological and pathological processes 66. CTB, which is normally expressed in cancer cells, is an inactive precursor that is only activated in lysosomes in an acidic environment, and then initiates a series of subsequent physiological reactions, including antigen presentation, protein degradation, and apoptosis. CTB is overexpressed in a variety of cancer cells, and significantly increases its activity, which then improves tumor invasion, angiogenesis, and metastasis 105. Therefore, developing probes that can accurately detect CTB activity and its biodistribution is of utmost importance for early diagnosis and treatment of cancer cells.

#### 2.8.1. Z-Arg-Arg and Z-Phe-Arg peptides

**Figure 5:**
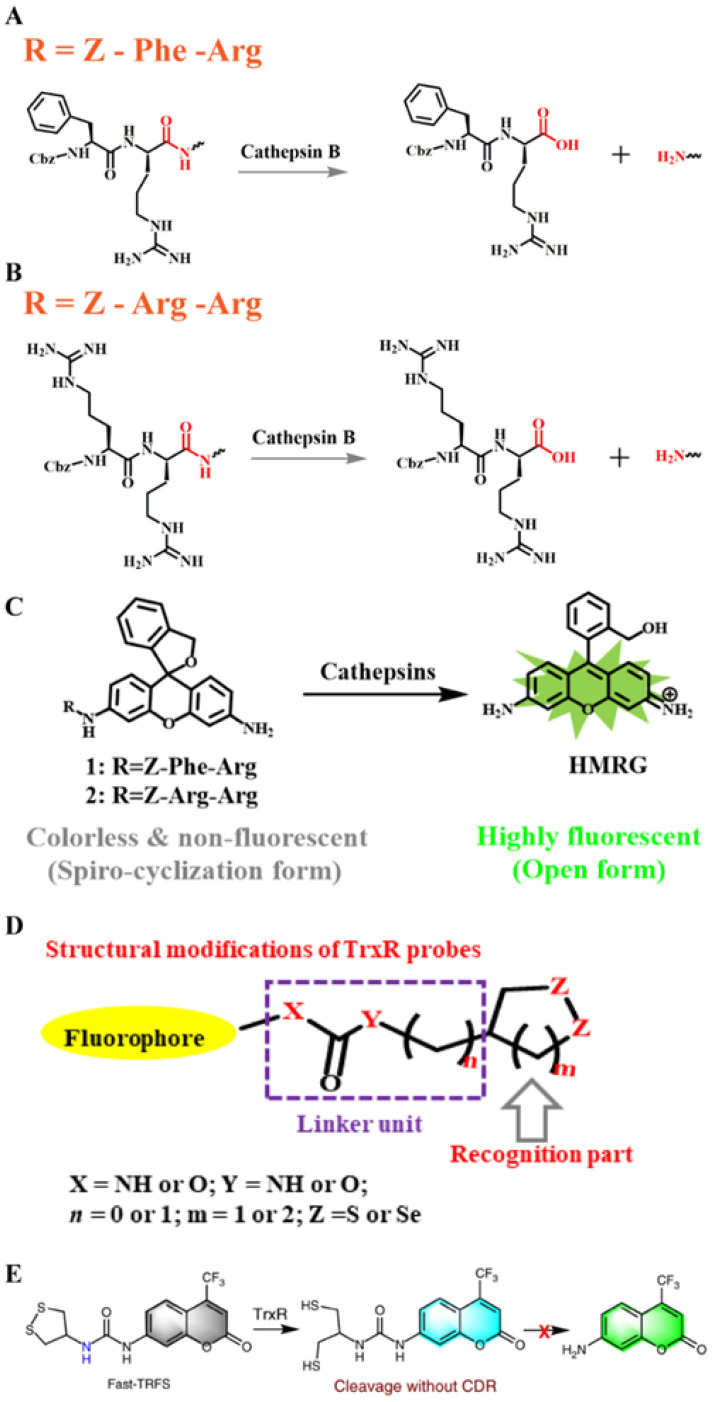
(A) Schematic illustration of the stimulation-response mechanism of CTB to the Z-Phe-Arg peptide; (B) Schematic illustration of the stimulation-response mechanism of CTB to the Z-Arg-Arg peptide; (C) Illustration of the FL probe responsive to CTB; (D) Schematic illustration of the stimulation-response mechanism of TrxR toward disulfides/diselenides; (E) Illustration of the FL probe responsive to TrxR. (C) Reproduced with permission 68. Copyright 2014, American Chemical Society. (D and E) Reproduced with permission 106. Copyright 2019, Macmillan Publishers Limited.

Both Z-Phe-Arg and Z-Arg-Arg peptide sequences can be recognized by and serve as selective targets of CTB enzymes (**Figure 5A**) 68. For example, CTB can catalyze the cleavage of the coupling moiety between Z-Phe-Arg or Z-Arg-Arg peptides and other molecules (**Figure 5B**). Based on this principle, a CTB enzyme-responsive hydroxymethylrhodamine green (HMRG)-based FL probe was successfully synthesized (**Figure 5C**). Under neutral conditions, the HMRG-based probe showed no FL. However, the FL intensity of released HMRG triggered by the enzyme showed an approximately 200-fold increase in FL. The experimental results proved that the HMRG-based probe can realize visual detection of human ovarian cancer cell lines SK-OV-3, SHIN-3, and OVCAR-3.

#### 2.8.2. GRRGKGG and KK peptides

In addition to the above-mentioned peptide sequences, other sequences, including Lys-Lys (KK) 60, Gly-Phe-Leu-Gly (GFLG) 65, GRRGKGG 66, and Val-Cit-Lys (Val-Cit-K) 69 have commonly been used as CTB recognition sites. However, the FL probes currently designed with KK and GFLG as selective targets are similar to Z-Arg-Arg (Z-R-R)- and Z-Phe-Arg (Z-Phe-R)-based probes, and show FL detection in a single activated visible light region. FL in visible light causes significant damage to tissue and the accuracy of the imaging results is greatly affected by the background signal. Fortunately, CTB sensitive NIR FL probes with GRRGKGG and Val-Cit-K as response sites have been successfully synthesized. Choi *et al*. designed a folate-targeting-CTB-specific activation probe using a sensitive group that has red-shifted the FL wavelength to the NIR region for achieving the imaging detection of live ovarian cancer cells 107.

### 2.9. Thioredoxin reductase (TrxR)-selective groups

TrxR is a pyridine nucleotide disulfide oxidoreductase, containing selenium cysteine residues. It uses the electrons provided by NADPH to maintain the reduced state of endogenous Trx, and further regulates numerous redox-related signaling pathways, including protein repair, transcriptional regulation, and antioxidant defense 108. TrxR dysfunction can lead to various diseases, including neurodegenerative diseases, inflammation, Parkinson's disease, and cancer 109.

Several disulfide/diselenide compounds can be employed as sensitizing substrates of TrxR (**Figure 5D**) 106. Disulfides/diselenides, as active response sites of TrxR, are convenient for detecting enzyme activity. A series of FL probes have been reported for detecting TrxR activity. For example, TrxR-green and TrxR-red FL probes use different colors of FL signals for detecting TrxR activity. Although the above-mentioned FL probes have good selectivity for TrxR, they still have limitations compared with the new type of Fast-TRFS probes. The Fast-TRFS probe provides a maximum FL signal intensity (approximately >150-fold increase in FL intensity) within 5 min of incubation with the TrxR enzyme. In addition, the Fast-TRFS probe displays higher selectivity to the TrxR enzyme compared to TRFS-red and TRFS-green. (**Figure 5E**). In brief, the probe uses a disulfide/dienamide bond to quench the performance of several fluorophores, as well as the disulfide/dienamide bond as a trigger site to achieve specific detection of enzymes and screen TrxR inhibitors via a simple and economical method.

### 2.10. Beta-galactosidase (β-gal)-selective group

β-gal is a type of exo-glycolase that selectively recognizes and cleaves glycosidic bonds in β-galactosides. In the body, β-gal breaks down lactose into glucose and galactose, thereby providing the body with the necessary carbon source and energy 112. Furthermore, β-gal is currently used in indirect treatment of lactose intolerance. By implanting the gene for β-gal in an organism's DNA via gene replacement therapy, individuals can autonomously undergo lactose decomposition reactions. Therefore, β-gal plays an important role in an organism's normal functioning. Surprisingly, in previous studies, it was shown that β-gal is significantly overexpressed in primary ovarian cancer. In addition, galactosidase is a biomarker for aging of the body's cells. As such, clear detection of β-gal activity and its location *in vivo* are essential for diagnosis and treatment of ovarian cancer.

**Figure 6:**
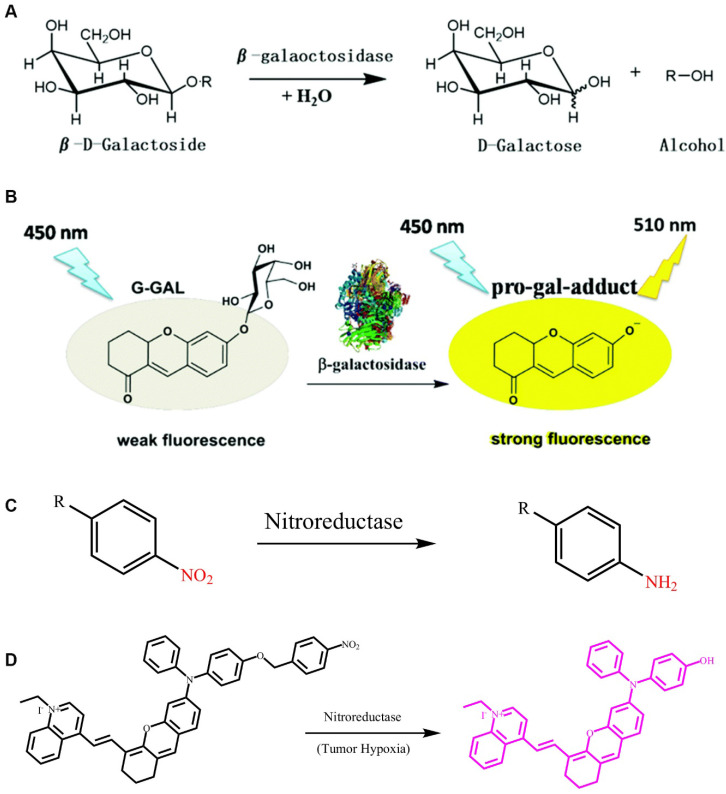
(A) Reaction mechanism of β-gal hydrolysis in organisms; (B) The FL light-up probe for β-gal detection; (C) Nitroreductase initiates the reduction mechanism; (D) The FL light-up probe for nitroreductase detection. (A and B) Reproduced with permission 110. Copyright 2019, The Royal Society of Chemistry. (D) Reproduced with permission 111. Copyright 2019, Wiley-VCH.

β-gal uses galactosyl as a substrate and hydrolyzes this into monosaccharides (**Figure 6A**) 110. Glycosidic bonds, which have a specific recognition site for β-gal, in galactose provide a selective target for the detection of β-gal *in vivo*. Based on this feature, related FL probes have been reported to track β-gal activity. In general, a FL probe is a β-gal-responsive FL probe that couples galactosyl to a chromophore containing a hydroxyl group. After the enzyme triggers hydrolysis of the glycosidic bond, the chromophore releases FL. However, most of these probes require a shorter excitation wavelength, which can lead to tissue damage, thereby limiting the application prospects of such probes. Fortunately, a class of two-photon-β-gal probes has been reported (**Figure 6B**) 110, in which xanthene-1-one (GCTPOC) is used as a two-photon chromophore to form an enzyme-responsive glycosidic bond with a galactosyl group to achieve controlled FL release. However, given the short emission wavelength of the probe, it was only used for the imaging and detection of enzymes in live cells, and thus greatly limiting the application of the probe. Thus, additional research for the design and development of FL probes for detecting β-gal is highly warranted.

### 2.11. Nitroreductase-selective group

Nitroreductase is a type of flavinase that can reduce nitro-substituted heterocyclic compounds and aromatic nitro compounds to aromatic amines 113. Based on their different electron transfer methods and sensitivity to oxygen, nitroreductases are divided into two categories: one type of nitroreductase follows the two-electron transfer method to reduce nitro compounds to amino compounds regardless of the presence of oxygen, whereas the other type can catalyze the reduction reaction of nitro groups only under anaerobic conditions 114. Previous studies have shown that the expression level of nitroreductase under hypoxic conditions is significantly increased in several tissues and tumors 115.

Nitro-aromatic compounds are the recognition group for nitroreductase, which exposes the self-eliminating group by catalyzing the reduction reaction of the nitro group (**Figure 6C**). As shown in Figure 6C, nitroreductase catalyzes the oxidation-reduction reaction of aromatic-nitro compounds, which promotes the conversion of electron-withdrawing nitro groups into electron-donating amino groups. Wu *et al*. developed a dihydroxanthene moiety and quinolinium as an electron donor-acceptor, and nitrobenzyloxydiphenylamino as the recognition group for nitroreductase. They further developed a highly sensitive response type nitroreductase optical probe (**Figure 6D**) 116. In this probe, the spectral characteristics of FL molecules were adjusted by constructing a D-π-A structure, which results in the probe having neither absorption nor FL in the NIR region when stimulated without nitroreductase. The nitro group in the probe molecule undergoes a reduction reaction to form an amino group in tissues where nitroreductase is overexpressed, thereby triggering a self-elimination reaction and releasing an activated probe molecule with FL and PA signals in the NIR region. The probe is not responsive to other endogenous substances in the cell, including reducing substances, such as glutathione and cysteine. This highly selective and sensitive FL probe enables accurate detection of the nitroreductase activity in live bodies, and provides a novel way for tracking both regional and distant breast cancer metastases that originated from orthotopic tumors via imaging.

## 3. Design mechanism of molecular probes for enzyme detection

In recent years, various enzyme-sensitive probes have been reported 117. These probes use enzymes to specifically recognize the performance of a reaction substrate and have various design mechanisms for enzyme detection, including in-situ self-assembly, intramolecular cyclization, FRET, ICT, and group self-elimination.

### 3.1. Signal conversion mechanism

#### 3.1.1. FRET

**Figure 7:**
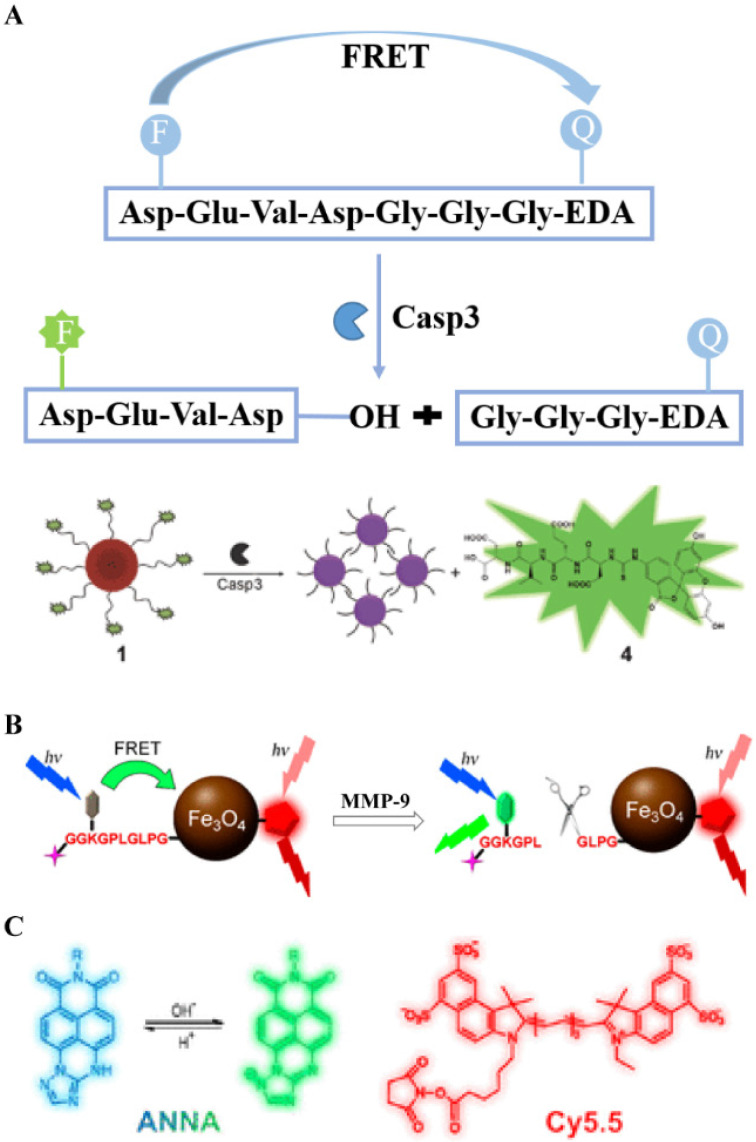
(A) Schematic diagram of caspase-3 releasing FL in response to DEVD peptide cleavage; (B) Mechanism of endogenous biological stimulation molecules triggering probe imaging; (C) Mechanism of pH-induced changes in FL signal of dye molecules; (A) Reproduced with permission 118. Copyright 2013, The Royal Society of Chemistry. (B and C) Reproduced with permission 15. Copyright 2017, American Chemical Society.

FRET characterizes a distance-dependent interaction in which an excited donor chromophore transfers energy to an acceptor molecule through non-radiative dipole-dipole coupling when two chromophores are close enough. In general, FRET is only effective when the distance between two FL molecules is less than 10 nm. In addition to the spatial distance requirement, the emission spectrum of the donor and the absorption spectrum of the acceptor must effectively overlap. During the FRET process, the FL intensity of the donor decreases, whereas that of the acceptor increases. Based on this mechanism, a variety of FL probes were designed to detect cancer-related enzyme activity. The FRET effect is based on the distance between donor and acceptor for efficient energy transfer, which makes it a powerful technique for studying the interactions of nano-biomacromolecules. Liang *et al*. employed Au nanoparticles and DABCYL as quenchers and caspase-3 reactive peptides as spacers to connect Au nanoparticles to the donor dye fluorescein isothiocyanate (FITC).^118^ The optical probe makes good use of the FRET mechanism (**Figure 7A**). Specifically, the probe uses an enzyme-response peptide sequence with a calculated length of 3.3 nm and a donor-acceptor unit that satisfies the spectral overlap conditions. The probe shows no FL in the absence of enzymes through energy transfer. However, after caspase-3-mediated substrate cleavage, FITC is released from the system, causing the FRET effect to disappear, and the FL intensity recovery to be promoted, which allows for the controlled detection of enzyme activity. In addition, electrostatic interactions occur between the released positively charged Au NPs, resulting in a severe agglomeration effect and a color change (red to blue) that is visible to the naked eye. Similarly, the MMP-9-responsive dual-ratiometric FL probe is also based on the FRET principle (**Figure 7B**). In this scheme, the presence or absence of chromophore FL before and after the enzyme reaction is controlled by the transfer of energy between the donor and the acceptor. The probe combines the FL signal of the chromophore that is activated by MMP-9 with the variable FL signal that is released by the pH-sensitive dye to form a dual-ratio FL probe for the quantitative detection of enzyme activity and pH in malignant tumors (**Figure 7C**). In general, the FRET mechanism is an important method for real-time, quantitative detection of various cancer-related enzymes.

#### 3.1.2. Enzyme-mediated ICT

An electron donor-π conjugated bridge-electron acceptor (D-π-A) molecule is formed by conjugation of an electron-donating moiety and an electron-withdrawing moiety into a π electron system. If ICT occurs, it will change the charge distribution of the entire system, leading to the dominance of the excited state of such D-π-A molecules. Formation of the ICT effect causes the polarity of the fluorophore to significantly increase, and eventually results in an extremely strong FL in both visible and NIR regions.

**Figure 8:**
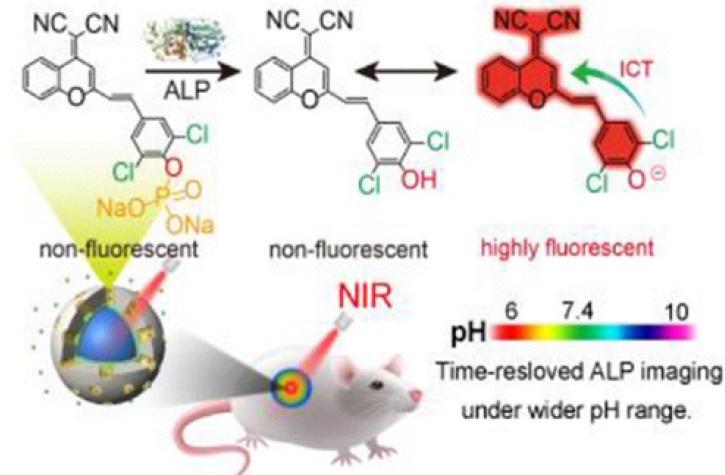
Schematic illustration of Cl_2_-BDCM-ALP for *in vivo* detection of ALP activity; Reproduced with permission 119. Copyright 2019, American Chemical Society.

At present, a series of FL probes designed using the ICT mechanism have been reported. These probes adjusted the π conjugation between the fluorophore and the analytes to change the signal intensity of the fluorophore, and allowed for real-time visual detection of tumors. Wei *et al*. used the ICT mechanism to prepare a red-NIR FL probe to detect ALP enzyme activity *in vivo* 119. They replaced benzene in dicyanomethylene-4H-chromene derivative (BDCM) with chlorine to obtain Cl_2_-BDCM, which is a stable and highly FL chromophore. Subsequently, the phenolic hydroxyl group in Cl_2_-BDCM was phosphorylated to create ALP-responsive FL probe (Cl_2_-BDCM-ALP). In the absence of ALP, Cl_2_-BDCM-ALP is non-FL. However, when ALP is present, Cl_2_-BDCM-ALP is dephosphorylated and the phenolic hydroxyl group in Cl_2_-BDCM exists as a phenoloxy anion, which triggers a rapid ICT effect, thereby leading to the FL of Cl_2_-BDCM-ALP (**Figure 8**). Thus, the enzyme-triggered ICT mechanism is an efficient strategy to develop responsive FL probes.

### 3.2. Probe activation mechanism

#### 3.2.1. Enzyme-triggered self-assembly

Self-assembly is ubiquitous in biological systems and can spontaneously form disordered particles into ordered supramolecular structures/nano-aggregates through non-covalent interactions, including electrostatic attraction, hydrogen bonding, and hydrophobic interactions. Self-assembly methods are used to assemble building blocks with various functions and activities into supramolecular structures/nano-aggregates, which can then produce complex biological material. In addition, during self-assembly, non-covalent interactions between particles can be adjusted to ensurethe stability of the assembly and the dynamic response of the assembly process.

**Figure 9:**
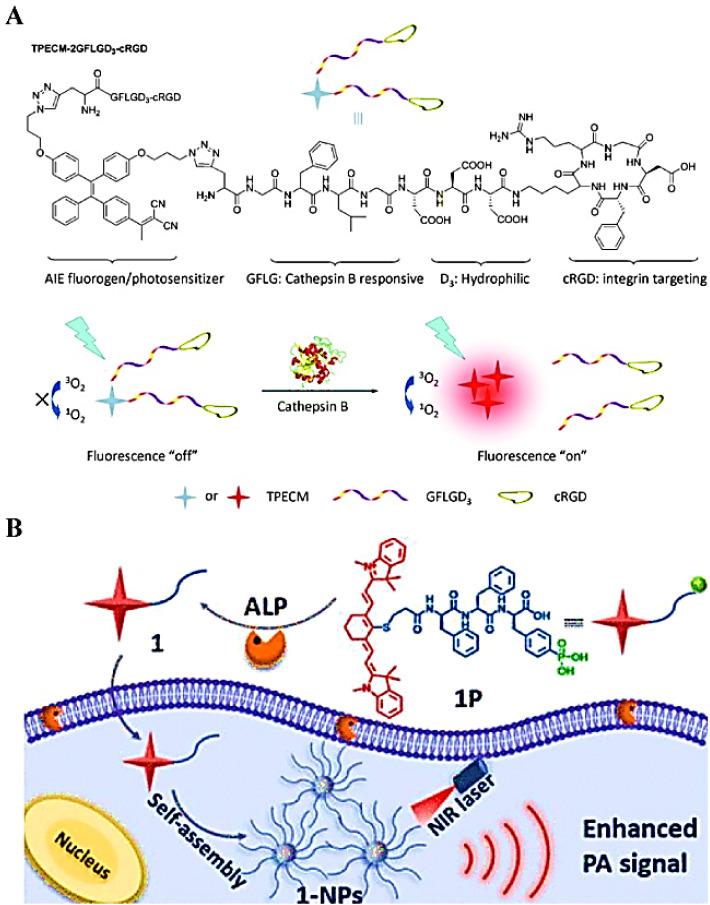
(A) Schematic representation of the chemical structure of the TPECM-2GFLGD3-cRGD probe activated by cathepsin B; (B) Schematic illustration of ALP-triggered self-assembly of NIR nanoparticles for enhanced PAI of tumor cells. (A) Reproduced with permission 120. Copyright 2015, Wiley-VCH. (B) Reproduced with permission 41. Copyright 2018, American Chemical Society.

Liu *et al*. used a CTB-triggered self-assembly strategy to selectively form nano-aggregates in cancer cells for imaging the enzyme activity in cancer cells 120. In their study, they combined a biocompatible polypeptide with the AIE molecule at a stoichiometric ratio of 2:1, to form a hydrophilic bioprobe (TPECM-2GFLGD3-cRGD) (**Figure 9A**). In the absence of CTB, AIE molecules were completely dissolved, and no FL emission occurred because of intramolecular rotation. Interestingly, the probe TPECM-2GFLGD3-cRGD was cleaved by CTB and released hydrophobic AIE molecules. Subsequently, these AIE molecules self-assembled into nano-aggregates to promote the activation of the probe. Similarly, alkaline phosphatase-mediated self-assembled PA probes were generated by dephosphorylation of the Phe-Phe-Tyr (H_2_PO_3_)-OH sequence, which increased the hydrophobicity, thereby leading to the spontaneous assembly of nanoparticles and enhancing the PA signal of the probe for tumor detection (**Figure 9B**).

#### 3.2.2. Enzyme-mediated self-immolate

**Figure 10:**
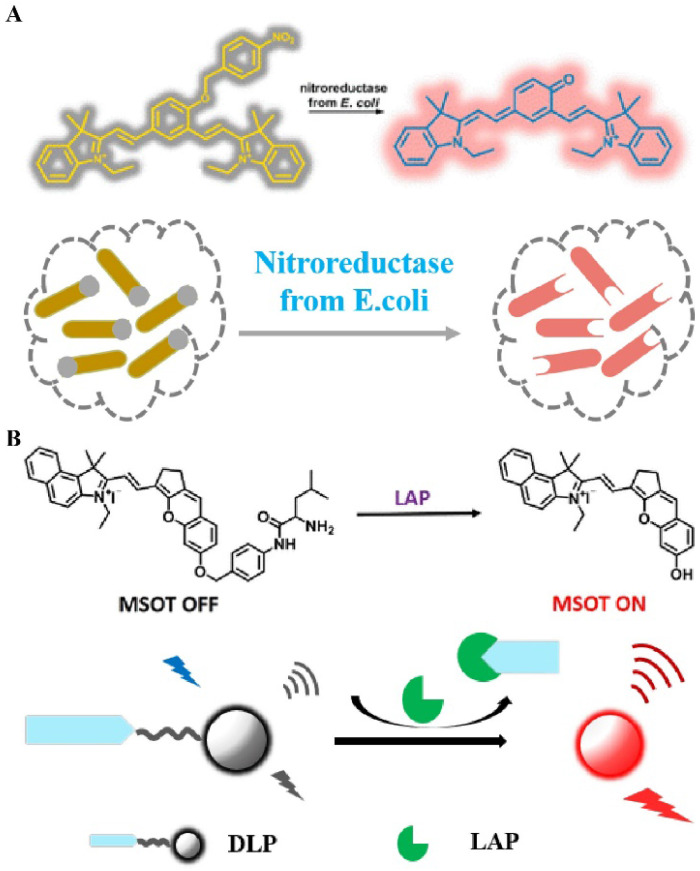
(A) Schematic illustration of nitroreductase selective activation of the bioprobe; (B) Schematic illustration of the chemical structure and *in vivo* imaging of the LAP activation probe (A) Reproduced with permission 122. Copyright 2016, American Chemical Society. (B) Reproduced with permission 123. Copyright 2019, American Chemical Society.

In general, combining certain specific groups with fluorophores causes the distribution of the electron cloud to change, thereby changing the emission wavelength of the FL dye or quenching the original FL signal. The application of this mechanism in combination with the enzyme trigger mechanism in the field of biomedicine is of great significance for accurately diagnosing the degree of tumor development 121. Therefore, a series of studies on enzyme-induced self-elimination of activated bioprobes have been reported. For example, Hecht *et al*. constructed an enzyme-activated FL probe by combining NIR FL dyes with nitroreductase-sensitive groups (**Figure 10A**) 122. After activation of the probe by nitroreductase, a two-electron reduction reaction occurs, which converts a strongly electron-withdrawing nitro group into an amino group. This triggers a self-elimination reaction, which in turn generates a carbonyl group of an electron donor, and activates a strong FL signal to achieve the accurate imaging of mitochondria. In addition to using the enzyme-mediated reduction reaction for triggering a self-elimination reaction to achieve FL recovery, the probe can also trigger self-elimination by cleavage to achieve enzyme-dependent signal regulation. A practical application of this phenomenon is observed in LAP-activated PA-FL probes (**Figure 10B**) 123, in which an enzyme recognition element (N-terminal leucyl moiety) is linked to chromene-benzoindolium (chromophore) through a self-eliminating group (4-aminobenzylalcohol group). Overexpression of LAP in the liver stimulates the cleavage of recognition fragments and subsequent self-elimination reactions, resulting in a red-shifted absorption band of the probe, which in turn leads to strong PA signals and NIR FL. In general, enzyme-sensitive units (peptide sequences, linkages, and functional groups) are combined with chromophores using self-immolative spacers, followed by enzymatic activation to trigger a cascade of reactions to achieve controllable signal regulation. These probes are important tools for detecting tumor-associated enzyme activity.

#### 3.2.3. Enzyme-induced macrocyclization

**Figure 11:**
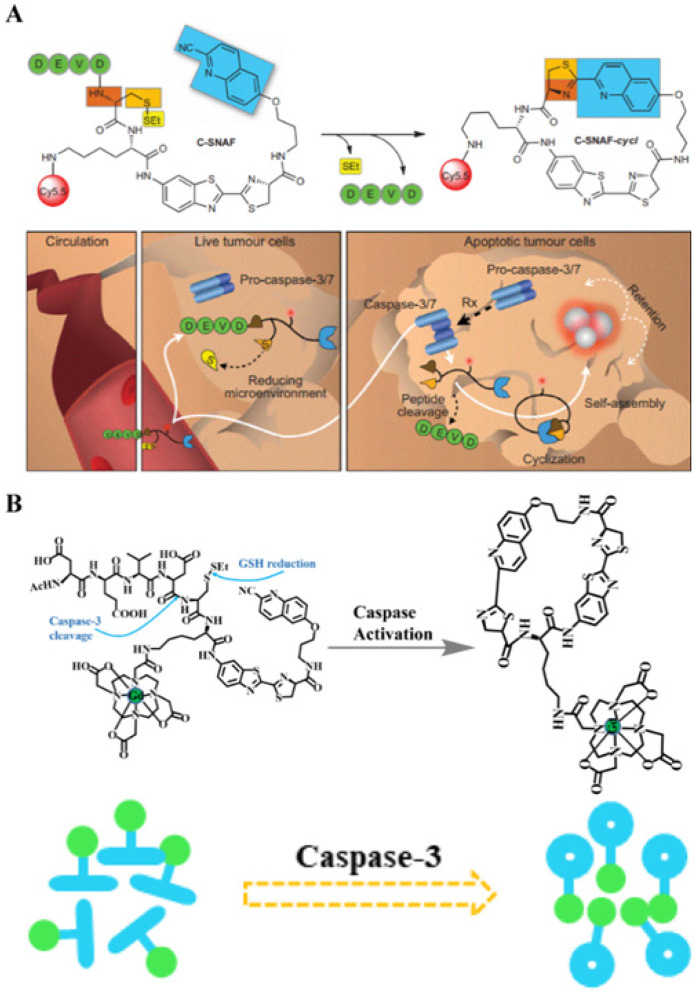
(A) Schematic illustration of *in vivo* imaging of caspase activity using the probe; (B) Illustration of caspase-3 triggering a Gd-based contrast agent cyclization process and assembly mechanism; (A) Reproduced with permission 124. Copyright 2014, Macmillan Publishers Limited. (B) Reproduced with permission 125. Copyright 2015, American Chemical Society.

The process of free groups in macromolecules undergoing rapid condensation within the molecule to form a cyclic compound is called intramolecular cyclization. In the field of biomedicine, molecular cyclization reactions are often accompanied with changes in other signals, including FL, PA, and magnetic resonance. Furthermore, if the molecule before cyclization is modified with a responsive moiety, analyte-specific activation of intramolecular cyclization can be achieved. Using this mechanism, Rao *et al*. prepared small-molecule probes that can image caspase activity *in vivo* (**Figure 11A**) 124. The probe consists of an L-DEVD sequence, an aminofluorescein molecule linked to D-cysteine and 2-cyano-6 carboxyquinoline (CHQ), and a disulfide bond group. The DEVD sequence in the probe is activated and cleaved by caspase to trigger a rapid intramolecular condensation reaction to form a circular molecule. Because the cyclic molecules are hydrophobic and rigid, they are susceptible to the influence of intermolecular interactions, (i.e., π-π stacking; hydrophobic interactions), which promote the *in situ* formation of aggregates by cyclic molecules. These aggregates are retained at the tumor site, and provide high FLI contrast for cancer detection. In addition to the imaging of changes in FL intensity caused by enzyme-mediated cyclization, caspase activity of apoptotic cells can be detected by changing the molecular magnetic resonance signal during the cyclization process. Similarly, an enzyme-sensitive DEVD sequence, a disulfide bond group, and a CHQ molecule are used as the probe backbone, and the cyclization reaction is triggered after the enzyme-responsive peptide is cleaved. In particular, a Gd-based contrast agent is attached to the probe backbone (**Figure 11B**) 125. Cyclized molecules accumulate in apoptotic cells and promote enhanced magnetic resonance signals for detecting enzyme activity. Therefore, combining the intramolecular cyclization reaction with enzyme response recognition provides a promising method for accurate cancer diagnosis.

## 4. Enzyme-activated cancer imaging

### 4.1. Enzyme-activated FLI

**Figure 12:**
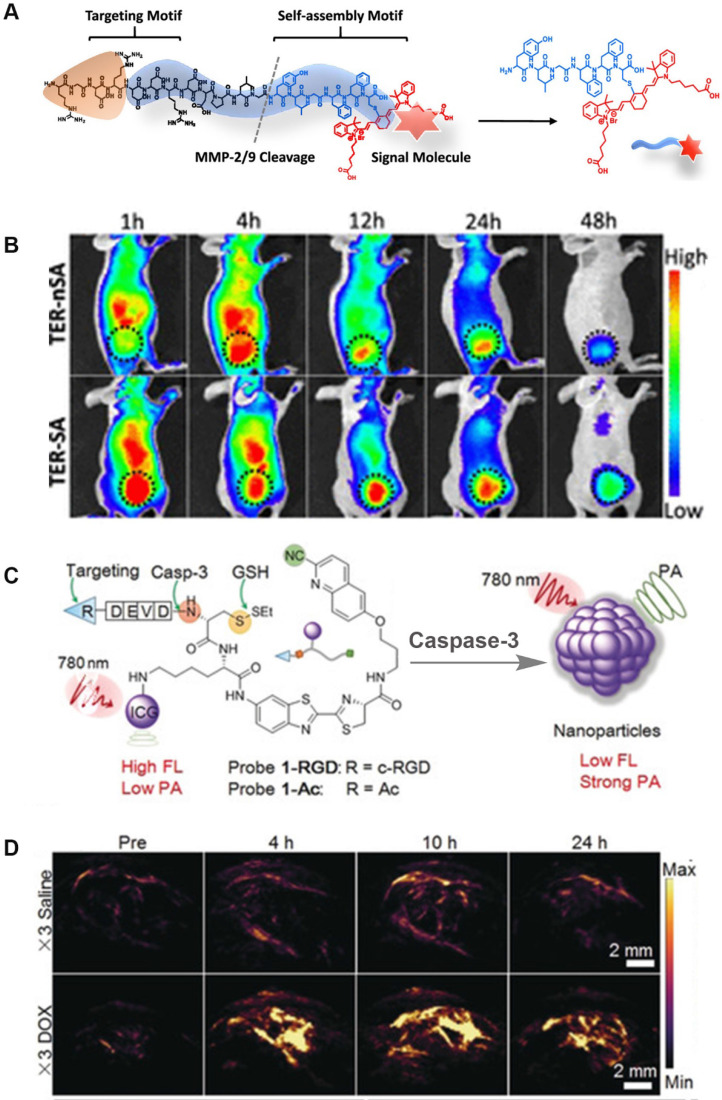
(A) The molecular structure of the probe; (B) Representative NIR FL images of TER-SA and TERnSA (3 mg/kg, n = 3) of 786-O-tumor-bearing mice after intravenous administration; (C) The chemical structure and chemical conversion process of the PA probe; (D) PA imaging signals in the tumor region at different time intervals after intravenous injection (top: PEG@CRUN, bottom: FA-PEG@CRUN, 3 mg in 100 mL saline); (A and B) Reproduced with permission 126. Copyright 2020, American Chemical Society. (C and D) Reproduced with permission. Copyright 2019, Wiley-VCH.

FLI, as an intuitive and efficient imaging method, is an important tool for monitoring disease development and for guiding the clinical application of surgery. It provides ultra-fast imaging of biological tissues during the detection process, and has high sensitivity and resolution. Considering the above-mentioned characteristics, a number of enzyme-sensitive FLI probes have been reported. For example, Xu *et al*. designed a NIR-peptide probe with a tumor-specific excretion-retarded effect for an imaging-guided surgery approach for renal cell carcinoma (**Figure 12A**) 126. In this probe, MMPs mediate peptide cleavage and trigger the spontaneous self-assembly of probe residues to form nanofibers, which result in the *in situ* enhancement of the signal-to-noise ratio in human renal cell carcinoma (RCC) tissue. Intriguingly, the assembled peptide (TER-SA) with a tumor-specific excretion-retarded effect (TER) shows a stronger FL signal when compared to the non-assembled peptide (TER-nSA) during *in vivo* detection at 12-48 h after injection administration (**Figure 12B**). In addition, the TER effect can accurately identify small lesions (<1 mm), helps complete tumor resection, and greatly reduces the recurrence rate after surgery. Overall, this type of FL probe that is based on the TER effect is of great significance for detecting tumor sites in metabolic organs.

### 4.2. Enzyme-activated PAI

As a new optical imaging method, PAI overcomes the limitation of FLI to detect enzyme activity *in vivo* because of its higher tissue penetration ability. More importantly, like FLI, PAI can achieve real-time quantitative determination of enzyme activity. Therefore, in recent years, enzyme-sensitive PA probes have been widely reported 127. In 2014, Liu *et al*. for the first time reported a novel activatable PAI nano-probe (CuS-peptide-BHQ3 (CPQ)) for *in vivo* detection of cancer-related MMPs. CPQ utilizes the characteristics of CuS and BHQ3 with specific absorption peaks at 930 and 680 nm, respectively, in addition to enzyme-responsive peptides to successfully detect MMP activity. Interestingly, in the absence of MMP, CPQ emits strong PA signals at 930 and 680 nm. In the presence of MMP, the PA signal at 930 nm is almost unchanged, however, that at 680 nm gradually weakens after cleavage by the MMP recognition peptide. This is mainly because the enzyme triggers CPQ to release BHQ3 and CuS nanoparticles, and the former being small molecules clear quickly. The experimental results demonstrate that the CPQ probe realizes the *in vivo* activity detection of specific enzymes for the first time via PAI. Gao *et al*. constructed a ratiometric PA probe (QC) that quantitatively detected MMP-2 activity using an enzyme-responsive peptide linked with a NIR FL dye (Cy5.5) and a quencher (QSY21) 14. Unlike CPQ, QC uses an enzyme trigger to break the FRET mechanism between Cy5.5 and QSY21 to achieve controllable adjustment of PA signal changes. In aqueous solutions, QC, as an amphiphilic molecule, tends to self-assemble into uniform nano-aggregates. After cleavage by the MMP-2 catalytic peptide, the hydrophilic unit Cy5.5 of QC is released and exists in free form, causing the light energy absorbed by QC to be partially released in the form of FL rather than heat, thereby weakening the PA signal. The probe only changes the PA signal under the induction of the enzyme response, while the PA signal remains unchanged under other stimulations, thus achieving the purpose of quantitative detection of MMP-2 activity *in vivo*. In a recent study, a caspase-mediated imaging probe for PA signal enhancement was reported (**Figure 12C**) 21. The ICG molecules in this probe emit weak NIR FL because of the aggregation-induced quenching effect. In addition, the occurrence of aggregation increases the non-radiative relaxation process, which significantly enhances the molecular PA signal. PAI results of caspase activity *in vivo* demonstrated that the blank control group and the experimental group showed weak PA signals before probe injection. After the probe was injected, the PA signal at the tumor site increased, and the maximum PA signal intensity was reached 10 h after injection (**Figure 12D**). In comparison, the PA signal intensity in the drug-treated group was approximately 4.4-fold higher compared to that in the saline-treated group. The above-mentioned findings indicate that enzyme-activated PAI is of great significance for early diagnosis and treatment of diseased tissues *in vivo*.

### 4.3. Enzyme-activated chemiluminescence (CHL) imaging

**Figure 13:**
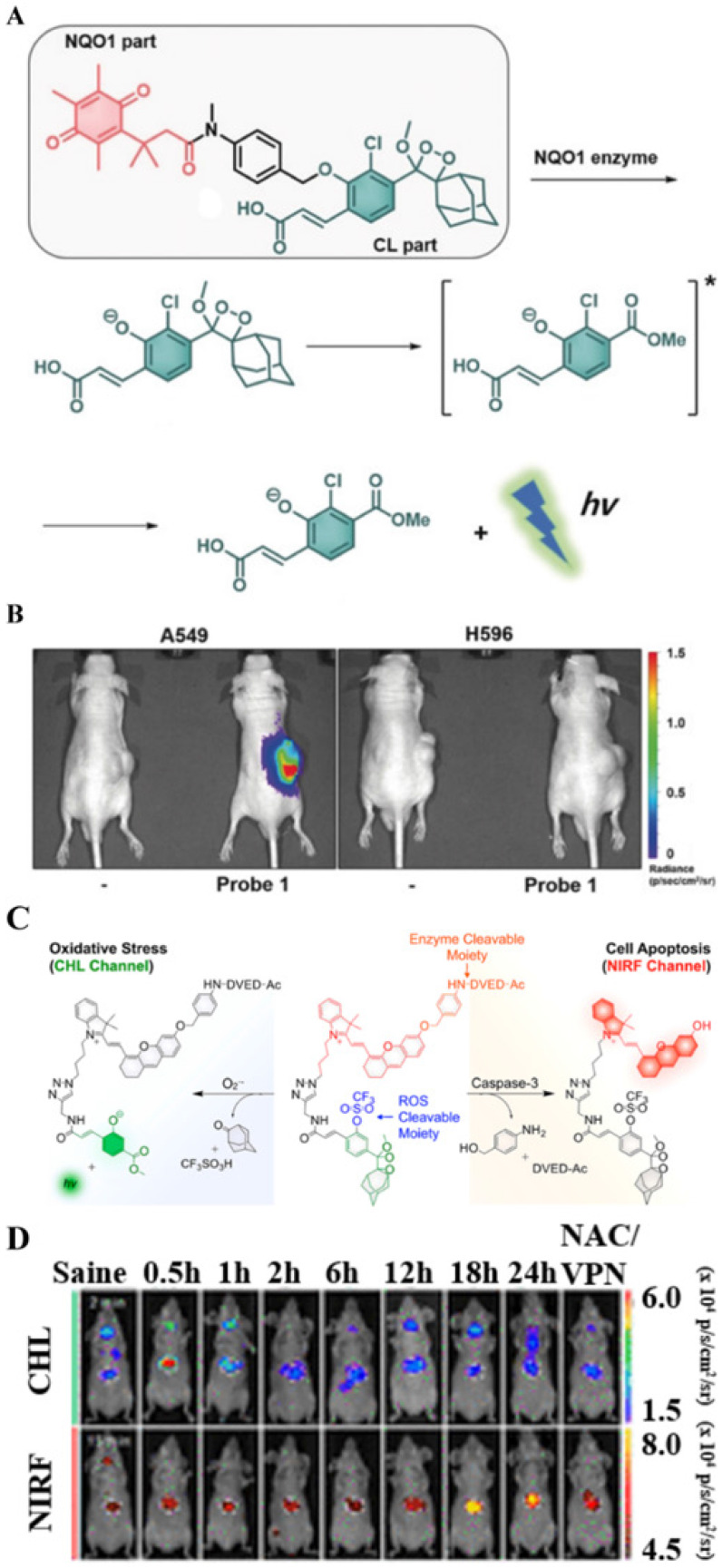
(A) The chemical structure of probe 1 and the mechanism of NQO1-triggered CHL release; (B) *In vivo* images of the xenograft model A549 and H596 cells obtained 10 min after intra-tumoral injection of probe 1; (C) The mechanism of endogenous molecules activating CFR to emit FL and CHL signals; (D) *In vivo* CHL and NIR FLI using probe CFR at 2 and 15 min after intravenous injection; (A and B) Reproduced with permission 47. Copyright 2019, Wiley-VCH. (C and D) Reproduced with permission 24. Copyright 2019, American Chemical Society.

Like FLI and PAI, CHL imaging is an optical imaging technique to image enzymes. However, CHL imaging does not require an external excitation light source, and tumor imaging can be achieved by the endogenous response to molecular stimulation, thereby greatly reducing the impact of external conditions. Therefore, CHL has broad prospects in detecting tumor-related enzyme activities with high specificity and high accuracy 55. A CHL probe (probe 1) was designed by Kim *et al*. 47 for imaging NQO1 overexpression in cancer. Probe 1 uses the self-immolative spacer para-aminobenzyl alcohol covalently linked with an acrylic-acid substituted phenoxy-dioxetane and a trimethyl-locked quinone (**Figure 13A**). Under physiological conditions, NQO1 triggers the deprotection of phenols, and the phenoxy-dioxetane part forms a phenolate, which spontaneously undergoes an excitation reaction to generate a green light. As shown in **Figure 13B**, a strong CHL signal was observed in the tumor region of A549-tumor-bearing mice. Corresponding NQO1-negative H596 cell-derived xenografts did not show a significant signal change. The *in vitro* and *in vivo* results were basically the same, thereby proving that NQO1 was overexpressed in the A549 cell line. Based on these findings, probe 1 can be applied in early diagnosis of cancers that overexpress NQO1.

The application of CHL probes in biomedicine has been extended. In a recent study, a chemo-FL-luminescent probe (CFR) that imaged liver toxicity was reported by Pu *et al*. The probe can simultaneously detect caspase-3 and O_2_^•-^ by independently responding to NIR FL and CHL (**Figure 13C**) 24. In this probe, caspase-3 selectively cleaves the DVED sequence to trigger the removal of the self-eliminating group from the molecule, thereby emitting NIR FL. At the same time, CHL is triggered by O_2_^•-^ to cleave the CHL group. The crosstalk-free duplex imaging method was designed for the real-time detection of two biomarkers involved in the process of liver toxicity. After intravenous injection of the CFR probe into the body, superoxide anion and caspase-3 overexpressed at the tumor site activated and released the corresponding optical signals, as shown in **Figure 13D** (CHL and FL images visualized 2 min and 15 min after CFR injection, respectively. The CFR probe uses two independent optical channels (enzyme-mediated NIR FL and O_2_^•-^-triggered CHL) for the successful early detection of drug-induced liver toxicity. Thus, both CHL imaging and NIR FLI are important tools for detecting cancer-related endogenous molecules.

### 4.4. Enzyme-activated bioluminescence imaging

**Figure 14:**
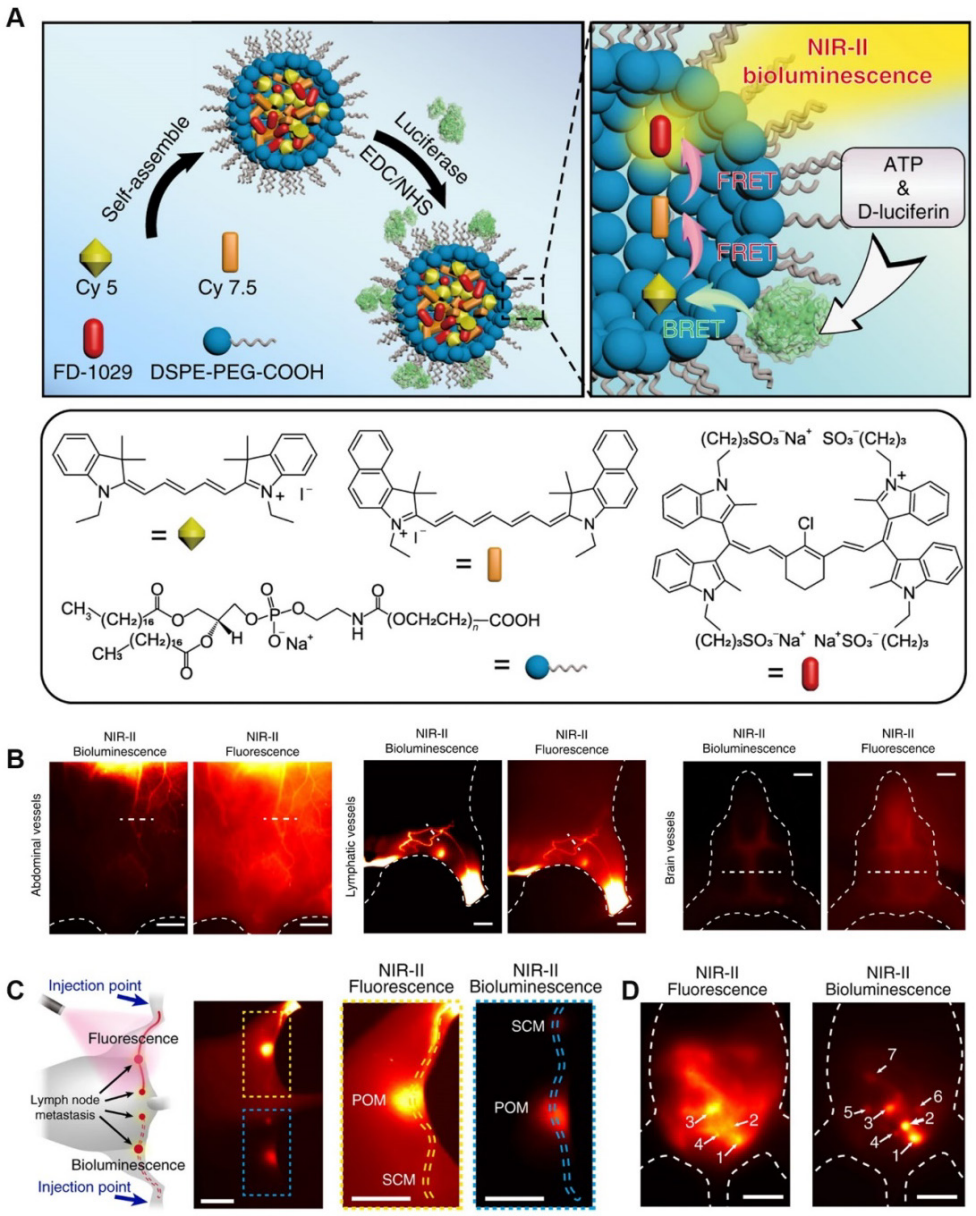
(A) Schematic procedures for synthesizing FD-1029 proble and mechanism of NIR-II BL; (B) NIR-II BL imaging (left) and NIR-II FLI (right) on abdominal vessels, lymphatics and brain vessels in mice after intravenous injection of FD-1029. (C) FLI (top) and BL imaging (bottom) of lymph node metastasis and the corresponding high magnification imaging (right). (D) Black dashed line indicates the Rose criterion. Reproduced with permission. Copyright 2020, American Chemical Society.

Bioluminescence (BL) without external excitation light, keeps the energy required for the luminescence process within the molecular structure, and releases it through a specific chemical reaction or enzyme-catalyzed reaction, thereby obtaining a bioluminescence signal 128. Due to its high sensitivity and high signal-to-noise ratio, BL imaging has been widely used in chemical and biological applications 129. Today, a variety of BL molecular probes have been developed to detect cancer-related endogenous biomolecules. A BL probe (FD-1029) was designed by Zhang *et al*. for imaging of vessels and lymphatics in mice **Figure 14A** 130. FD-1029 probe is a NIR-II BL realized by BRET and two-step FRET using specially designed cyanine dye. Subsequently, the FD-1029 probe was used to image blood vessels at different locations (abdominal vessels, lymphatics vessels and brain vessels)** (Figure 14B)**, and the results proved that the probe can clearly image the blood vessels at the cancer site. In addition, comparing FLI and BL imaging of the same part, the results show that BL imaging has a higher signal-to-noise ratio. The same result can be confirmed from the imaging results of live lymph node imaging (**Figure 14C**) and metastasis (**Figure 14D**).

### 4.5. Enzyme-activated magnetic resonance imaging

**Figure 15:**
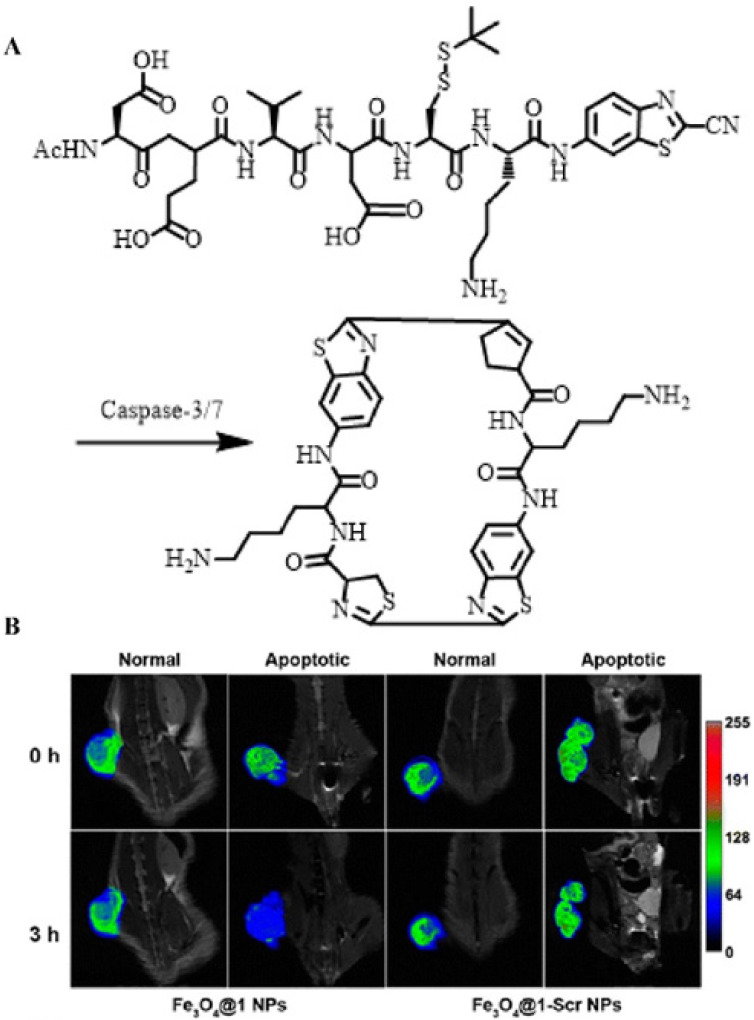
(A) Schematic illustration of caspase-3/7-instructed aggregation of Fe_3_O_4_@1 NPs; (B) T_2_-weighted coronal MRI of Fe_3_O_4_@1 NPs and Fe_3_O_4_@1-Scr NPs in saline-treated and DOX-treated mice; (B) Reproduced with permission 132. Copyright 2016, American Chemical Society.

Unlike optical imaging, MRI exhibited an ultra-high spatial resolution without external stimulation, which has also been extensively employed for enzyme detection 131. Therefore, the development of enzyme-responsively self-assembled MRI contrast agents is of great significance for the imaging and detection of malignant tumors. Based on this mechanism, Liang *et al*. constructed a caspase-indicated T_2_ MRI probe for monitoring the apoptotic process of tumor cells (**Figure 15A**) 132. They used an enzyme-triggered CBT-type condensation reaction to cause ultra-small paramagnetic iron oxide (USPIO) molecules to self-assemble into nano-aggregates to amplify the T_2_-type MRI signal intensity. When comparing the MRI results of Fe_3_O_4_@1 NPs (enzyme-triggered aggregation group) with those of Fe_3_O_4_@1-Scr NPs (non-response aggregation group) in both a normal mouse model and a drug-induced tumor apoptosis model, they observed that Fe_3_O_4_@1 NPs were effectively activated by over-expressed caspases to form assemblies during tumor apoptosis, thereby shortening the T_2_ relaxation time, and specifically enhancing T_2_-type MRI (**Figure 15B**). Such intelligent MRI probes regulated by enzymes provide a noval approach for the development and application of MRI.

### 4.6. Enzyme-responsive PET

**Figure 16:**
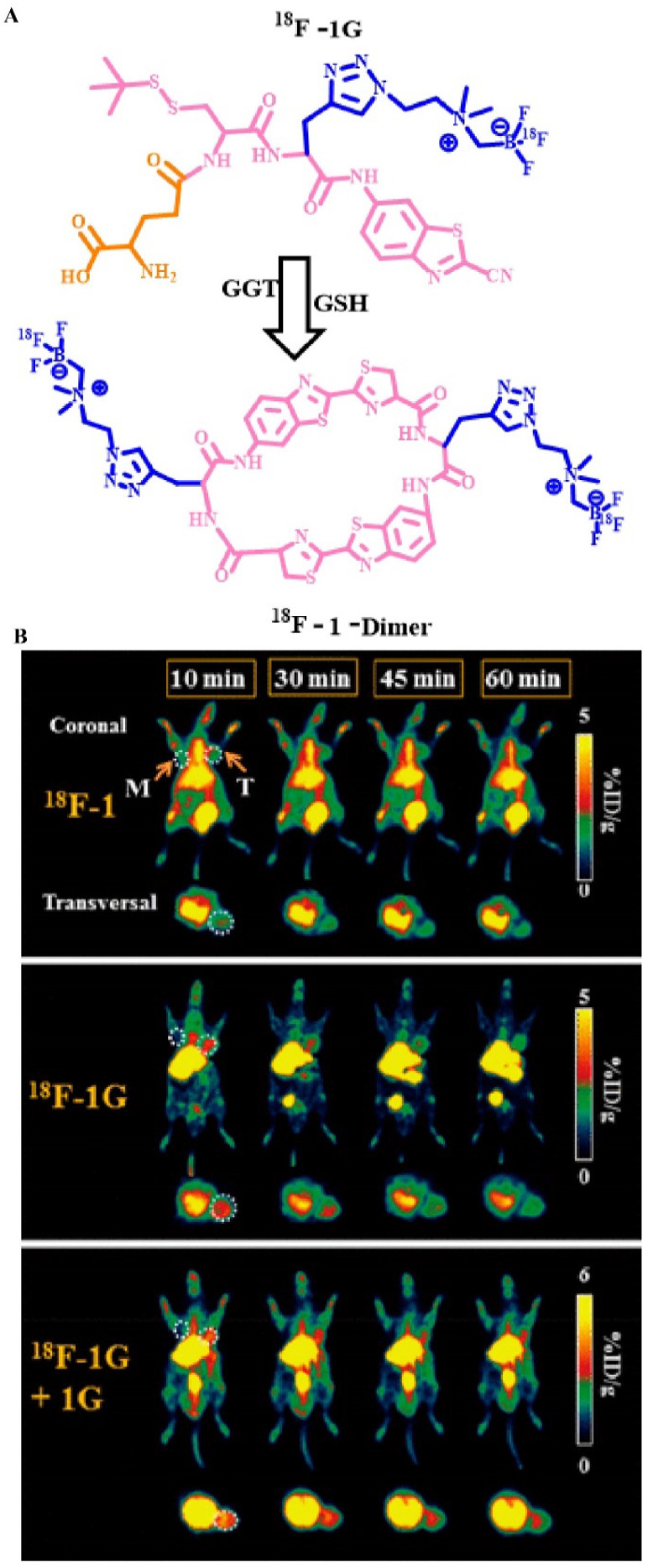
(A) The chemical structure of probe ^18^F-1G and the underlying mechanism by which GGT stimulates PET signal enhancement; (B) PET of GGT in HCT 116 tumor-bearing nude mice; (B) Reproduced with permission 52. Copyright 2020, American Chemical Society.

PET has a high sensitivity and specificity, which greatly improves its diagnostic accuracy. In recent years, in studies on the relationship between enzymes and tumors, PET technology has been used in the field of enzyme activity detection. For example, Lin *et al*. used fluorine-18 to label a γ-Glu-modified small molecule (**Figure 16A**) 23 and detected GGT activity *in vivo via* PET. They used a fluorine-18-labeled small molecule modified by γ-Glu [^18^F] γ-Glu-Cys (StBu)-PPG (CBT)-AmBF_3_ as a molecular probe (^18^F-1G) to monitor the process. Under physiological conditions, ^18^F-1G triggers the self-assembly of cyclic molecules into nano-aggregates through enzyme-specific response cleavage and reduction, which significantly amplify the PET signal. In addition, the uptake rate of the ^18^F-1G probe that was modified with a GGT-responsive matrix at the tumor site was approximately 2.7-fold higher than that of ^18^F-1 (without enzyme-triggered matrix). *In vivo* PET showed that ^18^F-1G, ^18^F-1, and ^18^F-1G + 1G (radiolabeled probes and non-radioactive precursors) had different imaging results at the tumor site (**Figure 16B**). The order of the PET imaging signal at the tumor site was as follows: ^18^F-1 < ^18^F-1G < ^18^F-1G + 1G. This order can be explained because of the higher uptake rate of molecular probes modified with enzyme response groups, the formation of aggregate states, and the longer retention time. In addition, the signal strength of ^18^F-1G was lower than that of ^18^F-1G + 1G on PET because the simultaneous addition of ^18^F-1G and 1G significantly improves the cyclization reaction and the subsequent self-assembly process. In general, the introduction of enzyme-sensitive units has greatly enhanced the signal strength of PET, which resulted in accurate monitoring of enzyme activity in related cancers.

### 4.7. Bimodal/multimodal imaging of enzymes

**Figure 17:**
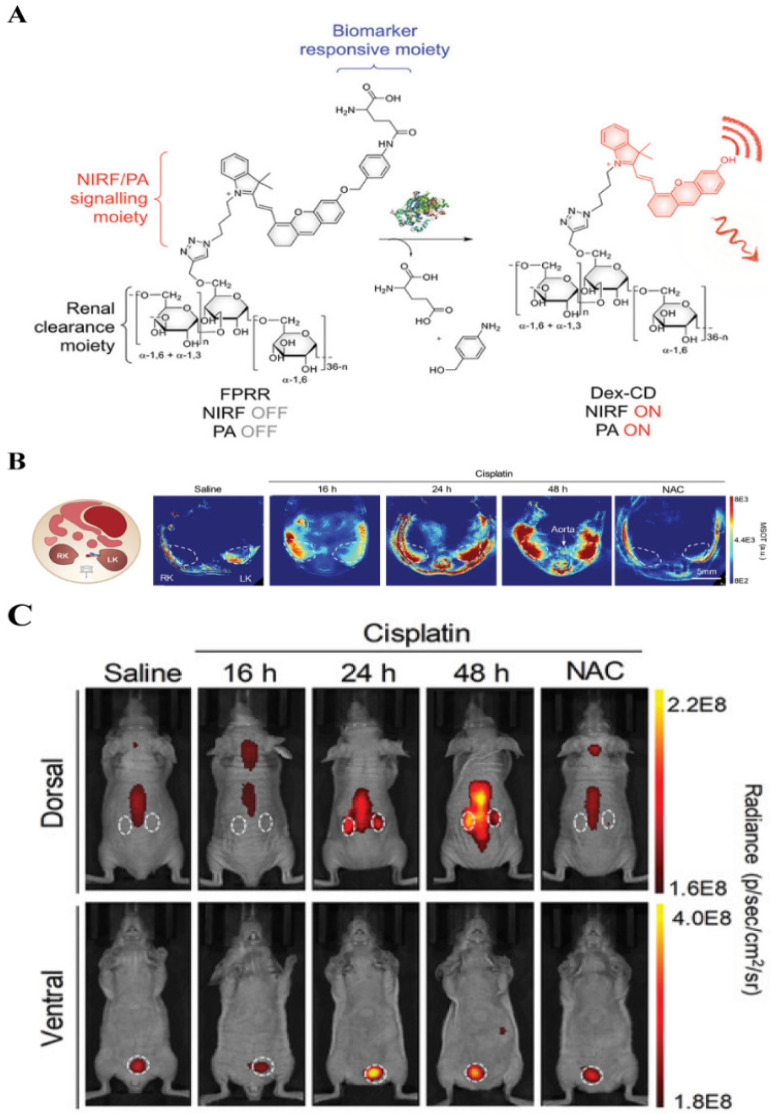
(A) Schematic diagram of FPRR in dual-mode imaging for acute kidney injury; (B) Representative PA images of mouse transverse sections at 2 h after intravenous injection of FPRR in different treatment groups; (C) Representative NIRF images of live mice at 60 min after intravenous injection of FPRR in different treatment groups; (A, B and C) Reproduced with permission 133. Copyright 2020, WILEY-VCH.

Dual-mode/multi-mode imaging solves the shortcomings of single-mode imaging by combining two or more imaging modes, and, simultaneously, provides high sensitivity, specificity, and penetration depth. Taking FL-PA dual-mode imaging as an example, it has been proven that the combined application of imaging methods can improve the accuracy of imaging results 8. Recently, Pu *et al*. reported on the use of a FL-PA polymer kidney receptor (FPRR) for real-time imaging of acute kidney injury FPRR has high sensitivity and specificity for γ-glutamyl transferase at the site of kidney injury *in vivo* 133. FPRR is stimulated by a high concentration of γ-glutamyl transferase at the kidney injury site, which can simultaneously turn on NIR FL and PA signal (**Figure 17A**). In addition, because of the high renal clearance of FPRR, acute kidney injury can be detected 24 h after drug treatment, which is 48 h earlier than conventional serum tests. In prior studies, *in vivo* imaging has shown that 60 min after FPRR injection, PA signals from activated FPRR could be observed in the pelvic and renal parenchyma on both sides of the aorta on multispectral optoacoustic tomography (**Figure 17B**). Furthermore, the NIR FL signal clearly shows the shape of the two kidneys (**Figure 17C**). Accordingly, as the first activated FL-PA probe, FPRR has enabled real-time molecular imaging of acute kidney injury, and thereby provided molecular guidance for the design of optics with high renal clearance, which is of utmost importance for clinical applications.

## 5. Conclusion and Perspective

In this review, we summarized the substrates (peptide sequences, linkers and groups) that can be selectively recognized by various enzymes, the design principles of enzyme-responsive probes, and the recent progress of enzyme-sensitive probes in the field of biomedical imaging. Depending on the characteristics that play a role in the recognition of reaction substrates by enzymes (for example, DEVD, IETD, and DDYVADC can only be cleaved by caspase-3/7, nitro reductase specifically reduces aromatic nitro groups, and NQO1 selectively catalyzes the cleavage of the amide bond in quinone propionic acid derivatives), a chemical structure that can be selectively recognized by the immobilized enzyme can be designed. Because of the specific mechanisms of action of different enzymes on exposure to the reaction substrate (e.g., caspases, MMPs, CTB, and other enzymes can only catalyze the cleavage of specific peptide sequences, nitroreductase promotes the redox reaction of the recognition group and ALP promotes the dephosphorylation reaction of the substrate), one cannot obtain the probe molecules required for enzyme activity by accurate detection through combining the contrast agent molecule with an enzyme-responsive structure. Therefore, contrast agent molecules were cleverly combined with enzyme-sensitive structural units to design responsive probe molecules for imaging detection of malignant tumors via mechanisms, including FRET, ICT, self-assembly, intramolecular cyclization, and self-elimination. So far, several enzyme-triggered imaging probes have been extensively studied, however, significant challenges remain for the quantitative detection of enzymes. This comprehensive review will provide important information to promote further development of enzyme activity detection probes.

Although some progress has been made in the detection of enzyme activity, many factors remain to be optimized. First, because of the complexity of the tumor microenvironment, the evaluation using most imaging probes is currently limited to *in vitro* studies, and real-time detection of enzyme activity *in vivo* has not been thoroughly studied. Therefore, accurate detection of enzyme activity *in vivo* in real-time and quantitative studies in the field of biomedical imaging are challenging. To solve the problem of real-time and quantitative detection of enzyme activity *in vivo*, the solution we propose is to design probes that can be combined with organelles, etc., to increase their enrichment time at the tumor site and extend the imaging time. Second, the currently used light excitation source for FLI and PAI contrast agents, and the detection signals released in the two imaging methods have a limited penetration depth, and can only achieve “visualization” imaging of subcutaneous tumors. Accordingly, real-time imaging of deeper tumors *in situ* remains a major challenge. In view of the limitations of the current imaging depth, the response probe in the NIR-II region can be designed to achieve deeper tumor imaging by reducing light scattering, reducing tissue absorption and reducing tissue autofluorescence background. Third, considering that under normal circumstances a malignant lesion often involves abnormalities in the activity of multiple enzymes, accurate, quantitative detection of multiple enzymes simultaneously is crucial. Finally, exploring the relationship between enzyme-related parameters and tumor evolution via imaging detection of enzymes is of great significance in early diagnosis and treatment of cancer. In order to be able to quantitatively detect enzyme activity *in situ* and establish a certain relationship between the expression levels of various enzymes and the evolution of tumors, to achieve early diagnosis of cancer. Our expectation is to combine standard signal molecules and response molecules to construct ratiometric imaging probes. Despite the immense challenges currently faced, the development of imaging probes that can quantitatively detect enzyme activity *in situ* is extremely important for early diagnosis and treatment of enzyme-related cancers, and will continue to be a research focus in the scientific community.

## Figures and Tables

**Table 1:** The substrates, characteristics/expression levels and detection methods of some cancer related enzymes.

Enzymes	Substrates	Characteristics / Expression level	Main detection methods
Caspases	DEVD 17	Caspase-3 is mainly overexpressed in tumor tissues 18, mainly including liver cancer, cervical cancer, colorectal cancer^19^, and glioma. The expression level of caspase-3 in tumor tissues is ~0.016 (caspase-3/β-actin), which is about 4-fold higher when compared to that in normal tissue (expression level of 0.004 caspase-3/β-actin) 20.	FLI 6, PAI 21, MRI 22, PET 23, CHL imaging 24, and PL imaging 3
	IETD 25		
	DDYVADC 26		
MMPs	GPLGVRGY 27	Expression levels of MMP-2 and MMP-9 are significantly elevated in most solid tumors, including breast cancer, bladder cancer, colon cancer, and prostate cancer 28. For example, the MMP-2 concentration in normal human serum is 593.3 ± 134.0 ng/mL, whereas the MMP-2 concentration in serum of breast cancer patients is 694.3 ± 140.5 ng/mL 29. The expression level of inactive platform MMP-2 (Mr: 72000) in normal cells and bladder cancer cells are 2.7 ± 0.6 units and 3.0 ± 0.5 units, respectively. Active species of MMP-2 (Mr: 59000 and Mr: 62000) are highly expressed in malignant tumor cells when compared with normal cells (1.9 ± 0.4 units versus 0.07 ± 0.05 units, respectively) 29. In bladder tissue, the concentration of MMP-9 is 1.7 ± 0.8 units in normal cells and 136.0 ± 4.0 units in bladder cancer cells 30.	FLI 31, PAI 32, and MRI 33
	GGKGPLGLPG 28		
	LGLAG 34		
ALP	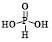 35	ALP is abnormally expressed in various cancers, such as liver cancer 36, prostate cancer, breast cancer, and bone cancer 37. The ALP level in normal adult serum is approximately 40-190 UL^-1^. The ALP activity range in 19 kinds of cancer cell line is 0.02-2157 nmoles MUP hydrolyzed per min per mg protein 38. [Lung cancer (SK-MES-1: 0.882; Calu-1: 0.280; SW-1271: 0.210); Ovarian cancer (SK-OV-1: 1.145; Caov-4: 86.71; SK-OV-3:0.153); Pharyngeal cancer (FaDu: 0.182); Breast cancer (SK-OV-1: 1.145; SK-BR-3: 53.80); Cervical cancer (HT-3: 93.13); Choriocarcinoma (JEG-3: 31.82); Colon cancer (HT-29: 0.520); Bladder cancer (T24: 0.695); Prostate cancer (DU 145: 0.234); Pancreatic cancer (Capan-2: 16.27); Kidney cancer (Caki-1: 0.038); Neuroblastoma (SK-N-SH: 2.01); Melanoma (HT-144: 0.057); Hepatoma (SK-HEP-1: 21.05); Osteosarcoma (Saoa-2: 2157.2); Diploid fibroblasts: 7.36±12.15; (units per mg protein)] 38.	FLI 39, MRI 40, PAI 41, and CHL imaging 42
	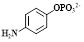 43		
NAD(P)H dehydrogenase [quinone]1 (NQO1)	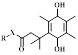	Compared with normal tissues, NQO1 expression levels are 5-200-fold higher in tumor tissues, including lung cancer, breast cancer, pancreatic cancer, head and neck cancer, and liver cancer 44. For example, in pancreatic tissue, the expression level of NQO1 in normal cells is 0.139 ± 0.024 (NQO1/beta-actin), whereas that in tumor cells is 0.831 ± 0.021 (NQO1/beta-actin) 45.	FLI 46 and CHL imaging 47
	X=N, NH, S, O 48		

GGT	γ-Glu 49	Transfected NIH-3T3 fibroblasts stably overexpressing GGT (~200-fold) 50. The level of GGT in human hepatoma and noncancerous tissues is 100.6 ± 87.4 IU/g protein and 46.6 ± 16.4 IU/g protein, respectively 51.	PET 52, MRI 53, FLI 54, and CHL imaging 55
LAP	LFGK 56	The normal LAP concentration is 130-380 units/mL, whereas the expression of LAP in liver cancer is 50 to 220 units 57.	FLI 58
	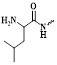 59		
Cathepsin B	Cbz-KK 60	The activity of CTB in cervical cancer cells is 23-fold higher compared to that in normal cervical epithelial cells 61. The serum CTB concentration in normal gastric tissue is 56.92 ± 67.4 pmol/L and in gastric cancer tissue is 129.41 ± 79.55 pmol/L (among them, diffuse type: 138.36 6 72.47 pmol/L; intestinal type: 109.16 ± 72.73 pmol/L) 62.	MRI 63 and FLI 64
	GFLG 65		
	GRRGKGG 66		
	Z-R-R 67 Z-Phe-R 68		
	Val-Cit-K 69		
CYP2J2	*O*-alkyl 70	CYP2J2 expression is elevated in human malignant cancers, such as esophageal, liver, breast, lung, and colorectal cancers 71.	FLI 72
Thioredoxin Reductase (TrxR)	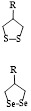 73	Compared with normal colonic mucosa, human primary colorectal cancer has 3-100-fold higher thioredoxin mRNA levels; thioredoxin reductase protein levels, and the activity in human colorectal tumor cells increased 2-fold on average. In human hematology and solid tumor cell lines, mRNA levels of thioredoxin and thioredoxin reductase increased by 10-fold and 23-fold, respectively 74.	FLI 75
Beta-galactosidase (β-gal)	β-galactoside 76	The activity of galactosidase in normal tissue is 0.06-0.17 μmol of product hydrolyzed/h/mg protein, and in ovarian cancer tissue is 0.03-0.474 μmol of product hydrolyzed/h/mg protein 77.	CHL imaging 78 and FLI 79
Nitroreductase	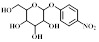 80	Hypoxia induces NTR overexpression in tumor tissues 81.	FLI and PAI 82
